# Association between autoimmune disease and neurodevelopmental disorder: a Mendelian randomization analysis

**DOI:** 10.1186/s13052-025-01910-2

**Published:** 2025-03-13

**Authors:** Jiangwei Qin, Yunfan Zhang, Ruolan Hu, Mingying Lin, Ruixin Yu, Yimin Hua, Yifei Li

**Affiliations:** Key Laboratory of Birth Defects and Related Diseases of Women and Children of MOE, Department of Pediatrics, West China Second University Hospital, Sichuan University, 20 3rd Section, Renmin S.Rd., Chengdu, 610041 Sichuan China

**Keywords:** Autoimmune diseases, Neurodevelopment disorders, Inflammation factors, Mendelian randomization, TWAS

## Abstract

**Introduction:**

Neurodevelopmental disorders such as attention deficit and disruptive behaviour disorders (ADHD), autism spectrum disorder (ASD), and schizophrenia have been increasingly prevalent recently. Previous research has demonstrated that inflammatory activity from autoimmune diseases is involved in neurological diseases. However, some studies question the association between inflammatory activities and neurodevelopmental disorders. Herein, we attempt to clarify this relationship using Mendelian randomization (MR) analysis.

**Methods:**

We used systemic lupus erythematosus (SLE), rheumatoid arthritis (RA), and type 1 diabetes mellitus (T1D) to represent autoimmune diseases. First, we conducted MR analysis to examine associated SNPs between autoimmune and neurodevelopmental disorders. Second, we performed bidirectional MR analysis to identify 429 types of signalling peptides and proteins or relevant receptors with causality reported diseases. Finally, we compared the genes with the gene loci identified in the available TWAS-hub site.

**Results:**

The MR results of autoimmune diseases on neurodevelopmental disorders did not present any significant association in all models. However, we identified 20–45 factors in ADHD, ASD, and schizophrenia, including semaphorin 3, IL-27 receptor subunit alpha, and fibroblast growth factor 16, which were considered clinically significant pro-inflammatory mediators. GO and KEGG enrichment analyses revealed unequal integrities among the three neurodevelopmental diseases, and we failed to identify a shared pathway linking autoimmune diseases and neurodevelopmental disorders. TWAS analysis indicated that CHRNA5 potentially mediates inflammatory activities in schizophrenia.

**Conclusion:**

According to our data, we failed to identify an association between autoimmune diseases and neurodevelopmental disorders. However, we demonstrated that some pro-inflammatory factors are involved in neurodevelopmental disorders.

**Supplementary Information:**

The online version contains supplementary material available at 10.1186/s13052-025-01910-2.

## Introduction

Psychiatric disorders, particularly neurodevelopmental disorders, have received significant attention from both the medical community and society in recent years. These disorders include a range of conditions such as anxiety, separation anxiety, intellectual disability, communication disorders, and learning disabilities, all of which negatively impact an individual's quality of life. Notably, the prevalence of newly identified cases of autism spectrum disorder (ASD) is increasing in both adults and children, especially in high-income countries [1]. The 2010 Global Burden of Disease study estimated that 52 million people were living with autism, resulting in a prevalence rate of 1 in 132 individuals [2].

Additionally, research involving children and adolescents across six continents and 35 countries found a national prevalence rate of approximately 5.29% for Attention-deficit disruptive behavior disorders (ADHD). Meta-analyses have revealed a worldwide prevalence rate of 2.5% among adults aged 19–45 years, with an estimated 15% of patients consistently diagnosed with ADHD in adulthood. This disparity between adult and pediatric prevalence suggests that developmental factors may contribute to the emergence of new cases [3, 4]. The incidence of schizophrenia is also concerning, with a global age-standardized time point prevalence of approximately 0.28% in 2016 [5]. These three prevalent mental health conditions have become focal points in the field of psychiatry [6, 7].

Although not fully understood, it is increasingly recognized that the inflammatory response may significantly influence neurodevelopmental disorders. These interactions are facilitated by shared anatomical features and signaling pathways, which include common receptors and signaling molecules. Evidence shows that immune regulation molecules, such as tumor necrosis factor (TNF), interleukin (IL) 1 beta, and various cytokines, along with their corresponding receptors, are present on sensory neurons [8, 9]. Additionally, receptors responsible for neural communication have been identified on immune cells in both the central and peripheral nervous systems [10]. Large-scale clinical studies provide compelling evidence of a connection between autoimmune diseases (ADs) and neurodevelopmental disorders. A previous systematic review revealed an increased risk of autism in children with a family history of ADs [11]. Similarly, both personal and maternal histories of ADs are associated with a higher risk of ADHD [12]. Moreover, a history of any AD has been linked to a 45% increased risk of schizophrenia [13]. Notably, between 40 and 90% of patients with systemic lupus erythematosus (SLE) develop neuropsychiatric symptoms, collectively referred to as neuropsychiatric lupus, which significantly affects their quality of life [14, 15]. In experimental studies, circulating IL-6 has been shown to bind to receptors on the vagus nerve, initiating a cascade of signals that trigger microglia-associated inflammatory responses [16]. Additionally, the cytokine-mediated responses of pathogenic microorganisms during host infections pose potential risks to neural systems [17]. Collectively, these findings suggest that the association between ADs and neurodevelopmental disorders may partly be due to shared genetic susceptibilities related to immune and cytokine-related genes associated with ADHD, ASD, and schizophrenia.

We propose that establishing connections with autoimmune diseases (ADs) can enhance our understanding of the pathogenesis of neurodevelopmental disorders. Initially, we sought to define a causal relationship between three common ADs—systemic lupus erythematosus (SLE), rheumatoid arthritis (RA), and type 1 diabetes mellitus (T1D)—and neurodevelopmental conditions such as ADHD, ASD, and schizophrenia, using Mendelian Randomization (MR) Analysis. Additionally, we systematically collected a comprehensive set of inflammatory factors to identify causal links among these six diseases and uncover the underlying signaling pathways. Our focus on molecules involved in intercellular communication is driven by our goal to explore the complex interplay between the immune and nervous systems. The ease of testing various circulating signaling molecules in the bloodstream allowed us to expand our dataset and improve our ability to establish connections between ADs and neurodevelopmental disorders.

The objective of this research is to synthesize information and establish causal associations between inflammatory responses and neurodevelopmental disorders. We aim to refine these associations by identifying and eliminating genetic sites associated with both ADs and neurodevelopmental disorders. The aim of this study was to provide compelling evidence that can inform subsequent mechanistic investigations and aid in the search for therapeutic targets. To achieve this, we will carefully identify specific loci within the immune system and neurodevelopmental disorders using a rigorous MR methodology. Additionally, we will include a comprehensive review of clinical literature and incorporate analyses using genome ontology (GO), Kyoto Encyclopedia of Genes and Genomes (KEGG), and transcriptome-wide association study (TWAS) techniques.

## Method

### Overview of the study design

This study aimed to identify potential causal relationships between autoimmune diseases (ADs) and neurodevelopmental disorders, including ADHD, ASD, and schizophrenia, and to validate potential genetic factors involved in immunologically related neurodevelopmental diseases. First, we performed MR analysis to determine whole-genome causality and identify associated SNPs between ADs and neurodevelopmental disorders. Second, we conducted MR analysis to identify inflammatory factors or relevant receptors with causal relationships to these diseases, based on data measuring the levels of involved molecules. Additionally, a literature review was conducted to identify potential inflammatory factors associated with ADs. Reverse causality was also considered to differentiate upstream and downstream relationships within specific signaling pathways by setting particular disease conditions as exposures for additional analysis. Furthermore, SNP-relevant genes were analyzed using variant effect prediction tools from Ensembl (ensembl.org/info/docs/tools/vep) to perform KEGG and GO enrichment analyses. Finally, we compared these genes with loci identified in available transcriptome-wide association studies (TWAS) analyses (twas-hub.org).

### GWAS data set

In line with our MR design, we focused on a cohort of six distinct diseases from the Finnish population sample (https://www.finngen.fi). It is important to note that information on the signaling factors was derived from a separate European population sample [18, 19]. Consequently, our MR analysis of ADs and neurodevelopmental disorders employed a one-sample design, while the assessment of relationships between signal mediators and these diseases used a two-sample design. Although a two-sample design may offer theoretical advantages, other available data sources were less complete compared to the comprehensive dataset from the Finnish population.

### Mendelian randomization

A Mendelian Randomization (MR) study must meet three prerequisite assumptions: (i) the instrumental variables (IVs) are correlated with the exposure; (ii) the IVs affect the outcome only through their effects on the exposure; and (iii) the IVs are independent of any confounders related to the association between exposure and outcome. Assumption (i) is under parameter control, while (ii) and (iii) are inherent but can be partially addressed by calculating pleiotropy and comparing different models. MR, which fundamentally involves a two-stage regression, is less susceptible to reverse causality and potential environmental or social confounding factors compared to clinical traits because it relies on genetic variants that are strongly and specifically associated with the exposure. The inverse-variance weighted (IVW) method with random effects was used as the primary MR analysis to examine the association between exposures (ADs or inflammatory regulation factors) and outcomes (neurodevelopmental diseases), including reverse causal analysis, with a significance level set at *P* < 0.05. For sensitivity analyses, MR-Egger regression, fixed IVW model, random IVW model, weighted median [20], simple mode [21], and weighted mode methods were also applied. MR-Egger regression was used to test for potential pleiotropic bias [22]. Heterogeneity was assessed using Cochran’s Q test on the IVW and MR-Egger estimates. Additionally, bidirectional MR analysis was conducted to explore the association between diseases and signal mediators [23, 24].

### Genetic instruments and parameters limitation

#### GO and KEGG analyses

For each of the six diseases (detailed in Supplementary Table 1), we identified SNPs with robust predictive capacity for the exposures, reaching genome-wide significance with a threshold of *P* < 1e^−5^. To minimize the influence of linkage disequilibrium (LD) and the superposition effect of correlated SNPs, we carefully examined LD patterns among the selected SNPs, retaining those with an r^2^ < 0.01. To ensure the suitability of instrumental variables and avoid weak instruments, we calculated average SNP-specific F-statistics. SNPs with F-statistics exceeding 10 were considered robust instruments for this MR analysis [25]. All results were assessed for pleiotropy, heterogeneity, and the number of standard-compliant SNPs. For the 429 signal mediators (Supplementary Table 1), the same parameters were applied when they served as exposures. The R package [TwoSampleMR] [26] was used for MR analysis (code provided in Supplementary file: code).

The extracted standard-compliant IVs were notably abundant, and most of the signal mediators in this research were linked to inflammation. Consequently, we performed GO and KEGG analyses using the statistically significant genes associated with the SNPs. These analyses aimed to eliminate redundant information and elucidate potential relationships between the immune system and neurodevelopmental disorders. We identified relevant genes through variant effect prediction tools within Ensemble-VEP. Notably, while most SNPs were associated with non-transcript loci, only a subset of SNP predictors yielded genes with demonstrable effects. Subsequently, we conducted gene clustering for GO and KEGG analyses using the R package [clusterProfiler] [27].

### Comparison of TWAS results

TWAS, or transcriptome-wide association study, is a method that integrates gene expression data with summary statistics from large-scale GWAS loci to identify genes whose cis-regulated expression is associated with complex traits of interest. This approach aims to identify more accurate and significant expression-trait associations than traditional GWAS [28, 29]. However, Yao et al. [30] found that, on average, only 9%−13% of heritability was mediated by gene expression levels, which cannot explain the majority of disease heritability. TWAS-hub, a relatively mature tool, was used with FUSION software, containing data on significant gene loci for various traits [31]. We compared the significant gene loci in our MR results with three available diseases (schizophrenia, lupus, and rheumatoid arthritis) in TWAS-hub.

## Results

### ADs did not contribute to the onset of neurodevelopmental disorders

**Fig. 1:**
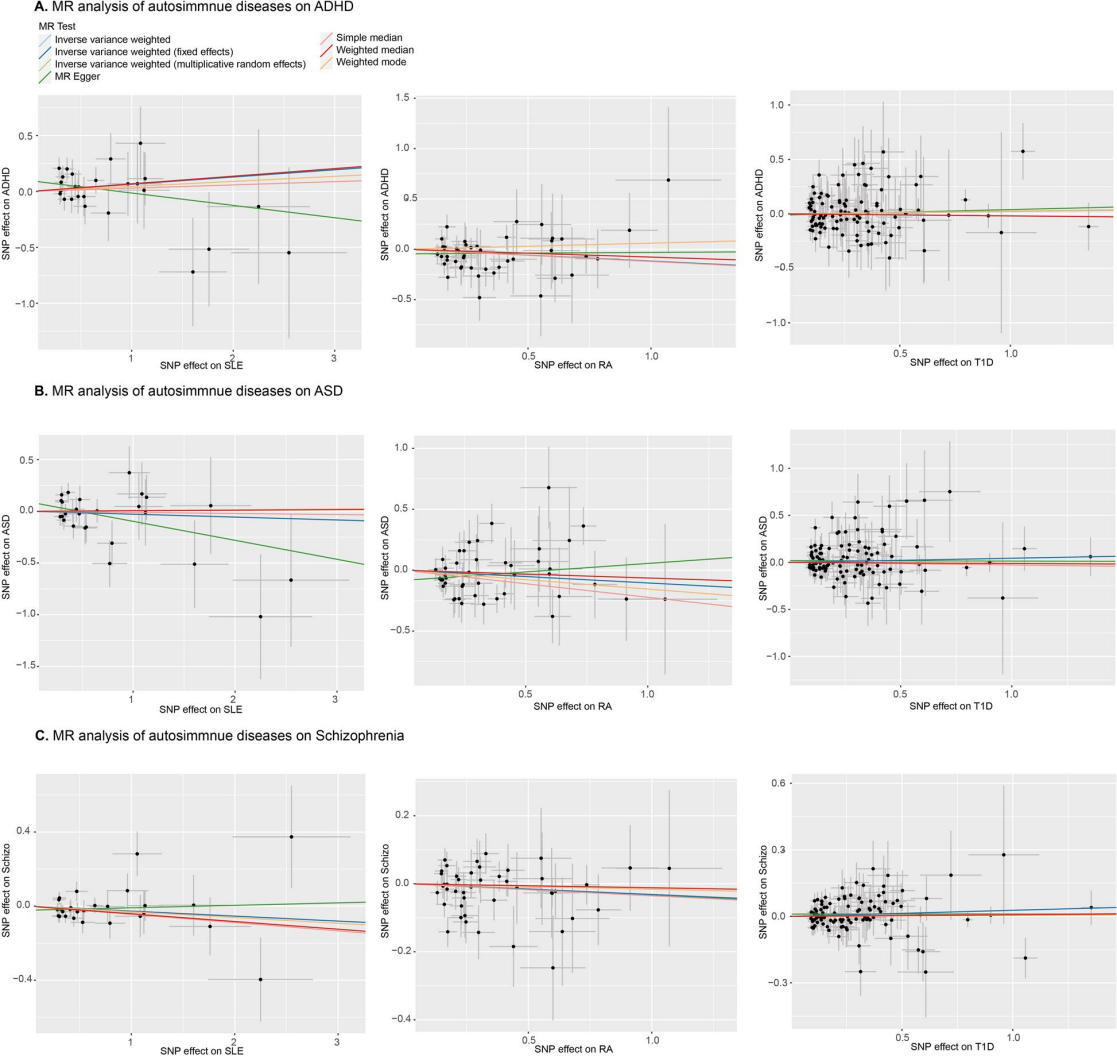
Two-stage regression plots of MR analysis of autoimmune diseases on ADHD, ASD and schizophrenia

One-sample MR analysis between autoimmune diseases (SLE, RA, and T1D) and neurological disorders (ADHD, ASD, and schizophrenia) was completed, suggesting a negative causal relationship among various MR models (including MR Egger, IVW (multiplicative random effects), IVW (fixed effects), Weighted mode, Simple median, and weighted median). The statistical significances of nine MR analyses for each parameter (Method: Inverse variance weighted) are summarized in Table 1. As noted, this analysis did not identify any causal association between autoimmune diseases and neurodevelopmental disorders. Two-stage regression plots are shown in Fig. 1.

**Table 1:** P-values of MR analysis between ADs and neurodevelopmental disorders (Method: Inverse Variance Weighted)

	SLE	RA	T1D
ADHD	0.251142	0.12285	0.59712
ASD	0.61645	0.178782	0.346315
Schizophrenia	0.198334	0.24188	0.093554

### Inflammatory regulation factors established the association between ADs and neurodevelopmental disorders

**Fig. 2:**
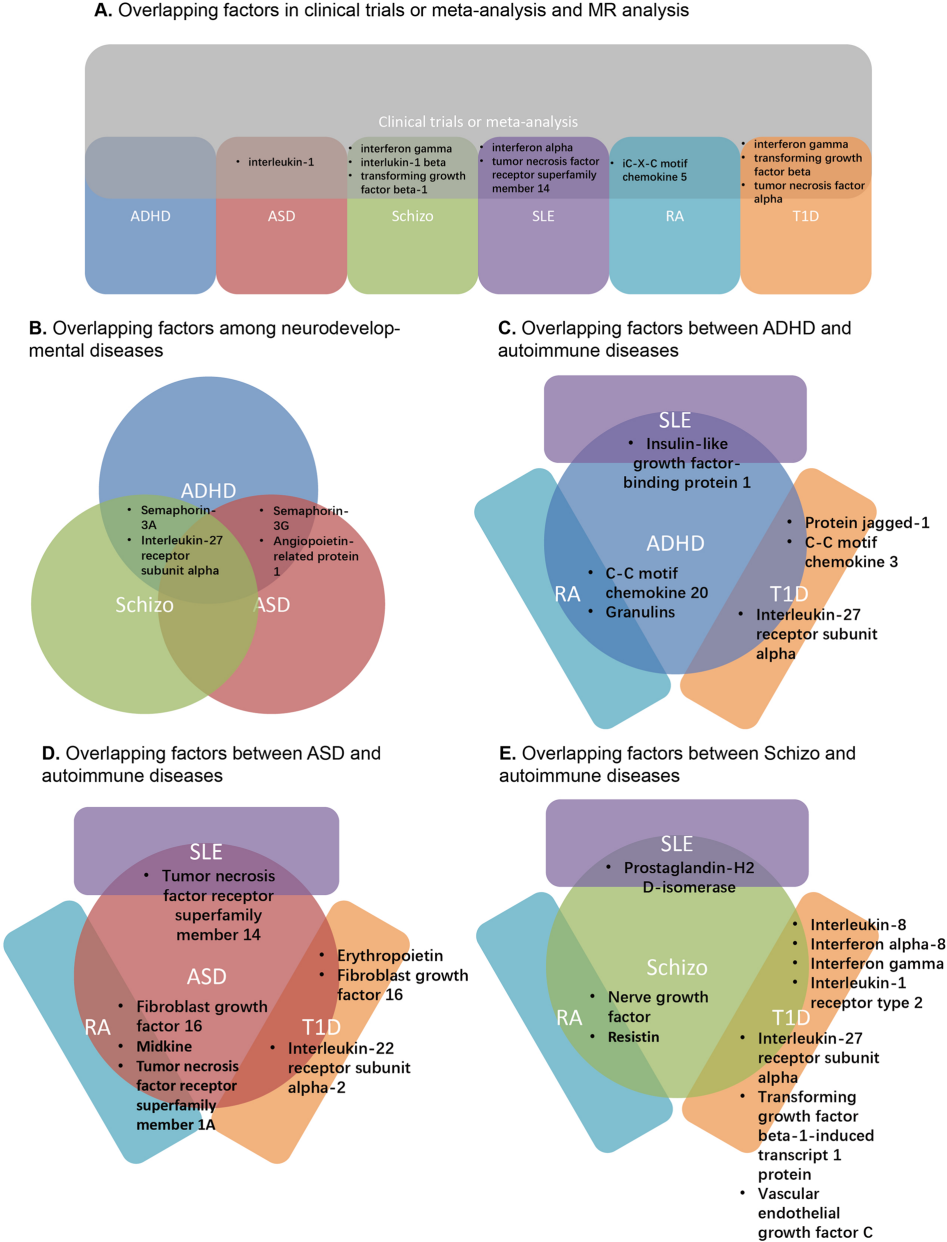
Summary of overlapping factors between clinical trials and MR analysis, and between two diseases

Bidirectional two-sample Mendelian Randomization (MR) was performed using 429 inflammatory regulation factors and the diseases mentioned earlier (SLE, RA, T1D, ADHD, ASD, and schizophrenia). A summary of all the inflammatory regulation factors involved can be found in Supplementary Table 2. These factors were initially retrieved from previous GWAS analyses focusing on genetic variants associated with these diseases. Clinical trait studies and related meta-analyses were systematically reviewed to identify potential inflammatory factors with statistical significance for each disease. The inflammatory factors identified through MR were then verified based on reported clinical traits. However, no validated factors were found for ADHD. IL-1 was confirmed to be associated with ASD, while Interferon (INF) gamma, IL-1 beta, and TGFβ−1 were identified as related to schizophrenia. Among the three types of autoimmune diseases (ADs), INF-alpha and tumor necrosis factor (TNF) receptor superfamily member 14 were associated with SLE. Additionally, C–C motif chemokine (CCL) 5 was implicated in RA in both MR and clinical trait analyses. INF-gamma, TGFβ, and TNFα were associated with the onset of T1D (Fig. 2A).

Furthermore, the potential overlap of identified inflammatory regulation factors between SLE, RA, T1D, and neurodevelopmental diseases was assessed (Fig. 2 B-E). IL-27 receptor subunit alpha was associated with three diseases (T1D, ADHD, and Schizophrenia), while fibroblast growth factor (FGF) 16 was identified in three diseases (T1D, RA, and ASD). Other inflammatory regulation factors were shared between only two specific diseases. Semaphorin-3G and angiopoietin-related protein 1 were involved in both ADHD and ASD. Bidirectional two-sample MR also revealed potential inflammatory regulation factors between ADs and neurodevelopmental diseases. Insulin-like growth factor binding protein 1 (IGFBP1) was identified in both SLE and ADHD. CCL20 and granulins were associated with RA and ADHD. IL-27, CCL3, and protein jagged-1 were involved in both T1D and ADHD. In ASD analysis, TNF receptor superfamily member 14 was related to SLE, while fibroblast growth factor (FGF) 16, midkine, TNF receptor superfamily member 1A, erythropoietin, and IL-22 receptor subunit alpha-s overlapped with RA and T1D, respectively. Interestingly, schizophrenia shared more inflammatory factors with T1D, including IL-8, INF-α8, INF-γ, IL-2 receptor, TGFβ−1, and VEGF. Additionally, prostaglandin-H2, nerve growth factor, and resistin, identified in the schizophrenia MR study, were also associated with SLE and RA.

### GO and KEGG enrichment analyses revealed unequal integrity among 3 neurodevelopmental diseases under the same conditions

**Fig. 3:**
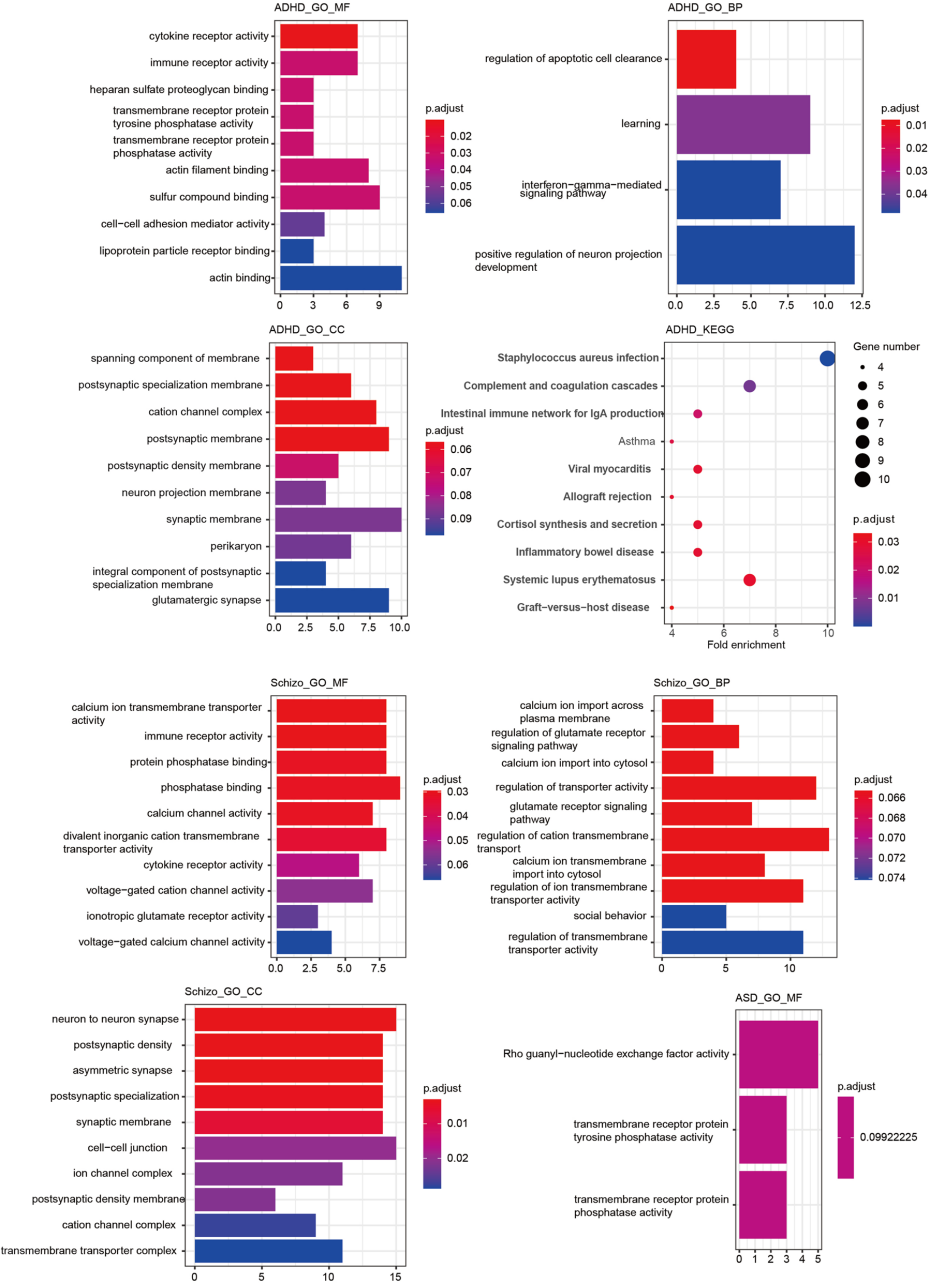
GO and KEGG enrichment analyses of significant SNPs-related genes of ADHD, ASD and schizophrenia

Significant SNP results from MR analyses of inflammatory regulation factors and six related diseases (including reverse analyses) were used to predict effects. The matching genes were then enriched in genome ontology and cellular pathway analyses. Figure 3 shows the top 10 potential inflammatory biological processes for ADHD, ASD, and Schizophrenia. ASD lacked KEGG, biological process (BP), cellular component (CC) results, and molecular function (MF). Supplementary Figs. 1–3 present enrichment analyses of genes associated with inflammatory regulation factor SNPs in ADs using the two-sample MR method. Table 2 provides a summary of common enrichment results (adjusted *p*-value > 0.05). KEGG pathways for ASD could not be completed due to limited targeted genes, and no specific enriched terms overlapped between patients with ADs and those with ASD. However, shared enriched terms, such as immune receptor activity, negative regulation of cell activation, and regulation of synapse organization, suggest that inflammatory activity is still involved in neurodevelopmental diseases.

**Table 2:** Summary of GO analyses between ADs and neurodevelopmental diseases

	**ADHD-T1D**	**ADHD-SLE**	**ADHD-RA**	**Schizo-T1D**	**Schizo-RA**
MF	➢ **immune receptor activity**	➢ **immune receptor activity**	➢ **immune receptor activity** ➢ transmembrane receptor protein tyrosine phosphatase activity ➢ transmembrane receptor protein phosphatase activity ➢ protein tyrosine phosphatase activity	➢ **immune receptor activity**	➢ **immune receptor activity**
CC	NA	NA	NA	➢ postsynaptic specialization	➢ neuron to neuron synapse ➢ asymmetric synapse ➢ synaptic membrane ➢ ion channel complex ➢ cation channel complex ➢ presynaptic membrane
BP	➢ negative regulation of cell adhesion ➢ **negative regulation of cell activation** ➢ **regulation of synapse organization**	NA	➢ regulation of cell morphogenesis ➢ cell–cell adhesion via plasma-membrane adhesion molecules ➢ neuron recognition ➢ synapse organization ➢ neurotransmitter-gated ion channel clustering ➢ glutamate receptor signaling pathway ➢ regulation of postsynapse organization ➢ **negative regulation of cell activation** ➢ modulation of chemical synaptic transmission ➢ regulation of trans-synaptic signaling ➢ postsynapse organization ➢ **regulation of synapse organization**	NA	NA

### TWAS analysis indicated dominant genes among diseases

TWAS-hub was involved in differentially expressed genes (DEGs) among schizophrenia, SLE, and RA. DEGs were then assessed for significance in MR analyses, and genes selected through this two-step process were considered as participants in specific diseases. For RA, ANKRD55, PTPN2, and PTPN22 were significant in both DEGs analysis and SNPs assessment from MR analysis. IRF5 was found to contribute to SLE. Similarly, CHRNA5 was identified as a risk gene for schizophrenia using the same analytical approach (Supplementary Table 3).

## Discussion

The parallels between the nervous and immune systems—particularly their components related to recognition and response to threats—have been studied for several decades [32]. Recent reviews have highlighted mechanisms underlying the interaction between neurology and immunology [33]. In neurodevelopmental disorders, peripheral inflammation and autoimmune disturbances can influence brain function. For instance, in Alzheimer's disease, misfolded and aggregated proteins may bind to pattern recognition receptors on microglia and astroglia, triggering an innate immune response characterized by the release of inflammatory mediators [34]. In the context of schizophrenia, anti-inflammatory pharmacotherapies such as aspirin, celecoxib, and minocycline have shown increased efficacy when used in conjunction with standard antipsychotic drugs, compared to using antipsychotic drugs alone [35]. Signals from the immune system can significantly influence the nervous system, and vice versa. For example, local inflammation can enhance the sensitivity of nociceptors in sensory neurons to harmful stimuli. This increased sensitivity is due to inflammatory molecules such as TNF, IL-1β, IL-6, and prostaglandins, which are released by various immune cells within the microenvironment [36]. Additionally, in autoimmune diseases, several neuro-modulatory immune mechanisms have been identified and are now part of clinical practice. Treatments for conditions like rheumatoid arthritis (RA) include anti-TNF agents, anti-IL-6 receptor antagonists, anti-CD20 antibodies, and T-cell co-stimulation inhibitors. However, these treatments carry risks of toxicity and adverse effects, making them unsuitable for some patients. A recent study demonstrated that a bioelectronic device designed for vagus nerve stimulation significantly improved the disease, presenting a promising alternative. This device also reduces TNF production [37], highlighting the potential of neuro-immune interventions in clinical management.

Despite numerous studies suggesting a potential link between autoimmune disorders (ADs) and neurodevelopmental disorders, our Mendelian Randomization (MR) analysis did not reveal positive results when considering ADs' instrumental variables (IVs) as exposures and neurodevelopmental diseases as outcomes. This indicates that any causal relationship is likely either indirect or relatively small from a genetic perspective. Consequently, we performed bidirectional MR analyses focusing on specific inflammatory cytokines. We screened approximately 20 to 45 factors for five diseases, excluding Type 1 Diabetes (T1D), resulting in 101 significant findings (see Supplementary Table 2). Overlapping outcomes across various neurodevelopmental disorders highlight three potential biomarker targets (see Fig. 2). Semaphorins 3 have been identified as key components in ADHD, ASD, and schizophrenia. Originally known for their role in guiding neural axon development, Semaphorins 3 are involved in processes such as neuronal proliferation, migration, neuritogenesis, and synapse formation. Notably, Semaphorin 3A is a major component of the extracellular matrix in neurons, enveloping specific types of neurons in the adult central nervous system (CNS) and contributing to neural plasticity. The binding of Semaphorin 3A (or other secreted Semaphorins) to chondroitin sulfate glycosaminoglycans may trigger signaling in specific brain regions. Semaphorin 3G plays a crucial role in regulating adult hippocampal connectivity and memory processes. This evidence suggests that Semaphorins are involved in regulating adult neuronal plasticity, offering new insights into how inflammatory cytokines impact neural damage [38, 39]. Angiopoietin-related protein 1 (ANGPTL1) is recognized as a key endogenous antiangiogenic agent that inhibits the proliferation, migration, tube formation, and adhesion of endothelial cells. It also affects cancer motility and may help preserve vascular integrity during local cerebral ischemia [40, 41]. Interleukin-27 Receptor Subunit Alpha (IL-27Rα) is the only significant shared element among ADHD, schizophrenia, and T1D. Its signaling influences various immune cells and can modulate either Th1 or Th2 responses. The Jak/STAT signaling pathways activated by IL-27Rα ligation are related to inflammation [42]. These findings highlight potential biomarkers and pathways that could help elucidate the complex relationship between inflammatory cytokines and neurodevelopmental disorders.

Regarding the overlap between ADs and ADHD, there are unexplored relationships that warrant further investigation. Granulin, a small cysteine-rich peptide, is produced through the proteolytic cleavage of progranulin. Research has suggested that progranulin helps suppress aberrant microglia activation during aging [43]. Variants of the Jagged-1 protein are associated with Alagille syndrome, which causes developmental abnormalities in organs such as the liver and heart. However, no documented links between this protein variant, AD, and neurodevelopmental diseases have been reported [44]. CCL3 and CCL20 act as chemoattractants for specific immune cell types. IGF-I exhibits anabolic, antioxidant, anti-inflammatory, and cytoprotective properties, primarily dependent on growth hormone, although not exclusively [45]. These findings suggest potential avenues for further research to uncover the intricate relationships between these factors and their possible involvement in the association between AD and ADHD [45].

In the context of the interaction between AD and ASD, several common underlying characteristics involve inflammatory regulation factors, including Midkine, erythropoietin, FGF, TNF, and IL-22 receptor. Midkine contributes to cell proliferation by interacting with anaplastic lymphoma kinase (ALK) and participating in neointima formation following arterial injury [46]. Erythropoietin stimulates both proliferation and differentiation of cells. FGF16 is associated with proliferation-related processes, particularly in heart development [47]. The identification of TNF receptor superfamily and IL-22 receptor provides evidence of immune cell involvement in the complex interplay between AD and ASD. The shared characteristics and mechanisms underlying the relationship between AD and ASD warrant further investigation to better understand their potential connections.

In the context of schizophrenia, our research has identified several noteworthy associations with inflammatory regulation factors. Prostaglandin-H2D-Isomerase plays a crucial role in the transformation of prostaglandins, a key step in their maturation [48]. Resistin, a pro-inflammatory molecule, regulates various chronic inflammatory diseases, metabolic disorders, infectious ailments, and cancers [49]. TGFβ−1 is involved in a range of physiological and pathological processes, including cell cycle arrest, cell proliferation, cell differentiation, extracellular matrix production, immunosuppression, and oncogenesis [50]. Inflammatory factors such as INFs and ILs are implicated in the complex pathophysiology of schizophrenia. These findings provide insights into mechanisms and factors that may enhance our understanding of the relationship between inflammation and schizophrenia.

Based on this comprehensive overview, it is evident that while inflammatory signaling significantly mediates neurodevelopmental disorders, other factors such as microglia condition, vascular endothelial cell stability, and cell proliferation pathways may also play roles in these interactions. However, inflammatory regulation factors appear to be distributed unevenly across these neurodevelopmental diseases. Despite conducting extensive bidirectional MR analysis with numerous signal mediators, we could not identify a predominant pathway shared among ADHD, ASD, and schizophrenia. This observation corresponds with the unequal enrichment analysis results among these conditions (Fig. 3).

Upon analyzing data from TWAS-hub, CHRNA5 emerged as a significant gene mediating the relationship between schizophrenia and inflammatory activity. Although CHRNA5 research has primarily focused on smoking-induced diseases and nicotinic acetylcholine pathways [51–53], Fani et al. [54] identified associated schizophrenia symptoms linked to genetic loci related to CHRNA5. Notably, decreased activity in CHRNA5-altered mice mirrors the reduced prefrontal cortex activity seen in patients with schizophrenia and addiction. Nicotine also differentially affects related sites. Importantly, CHRNA5 was highlighted through variant effect prediction of rs56160480, an instrumental variable in our MR analysis linked to transforming growth factor alpha (GWAS ID: prot-a-2961). This variant is located within an intron, suggesting that TGF-alpha may influence schizophrenia development through non-transcribed mechanisms.

While our primary focus was on peripheral autoimmune elements, it is important to acknowledge research exploring psychiatric symptoms associated with autoimmune diseases (ADs). These studies suggest that autoimmune conditions may primarily involve factors within the central nervous system (CNS) rather than just peripheral ones. For example, lupus mice have been observed to develop increased anxiety-like behavior and sustained phagocytic microglia reactivation before showing any clear peripheral lupus pathology. Microglia might play a crucial role in coordinating synaptic loss and supporting neurons [55].

In summary, we have provided a partial synthesis of associations derived from scientific disciplines, including epidemiology and genetics. However, certain limitations in our study should be noted. First, our study population was limited to European groups, and SNP distributions can vary significantly among different racial groups. Second, data constraints prevented us from comparing transcriptome-wide association study (TWAS) results for autism spectrum disorder (ASD) and attention-deficit/hyperactivity disorder (ADHD). Additionally, the inflammatory signal mediators were selected from terms categorized under “Inflammation Mediators” (ncbi.nlm.nih.gov/mesh/68018836) and “Intercellular Signaling Peptides and Proteins” (ncbi.nlm.nih.gov/mesh/68036341) as integrated by NCBI Mesh Terms. While we aimed to include nearly all relevant molecules, it was not possible to account for all associated molecules due to incomplete GWAS summary data from the “IEU GWAS Open Project” (gwas.mrcieu.ac.uk/).

## Conclusion

As a result, our current data does not provide a comprehensive understanding of the associations between immune activity or inflammatory responses and neurodevelopmental disorders. Instead, it highlights the need for further in-depth exploration of the existing evidence. Nevertheless, Semaphorin 3 and the CHRNA5 gene emerge as promising candidates for potential breakthroughs in this area of research.

## Supplementary Information

Supplementary Material 1: Supplementary Fig. 1–3. GO and KEGG enrichment analyses of significant SNPs-related genes of SLE, RA and T1D.

Supplementary Material 2: Supplementary Table 1. GWAS-ID with name information.

Supplementary Material 3: Supplementary Table 2. MR results and clincial traits.

Supplementary Material 4: Supplementary Table 3. TWAS-hub match results.

## Data Availability

All the data had been presented in the manuscript. Other data sets used in this study are available from the corresponding author upon reasonable request.
