# Supplementary figures and images for: Association between autoimmune disease and neurodevelopmental disorder: a Mendelian randomization analysis
Source: Ital J Pediatr. 2025 Mar 13;51:76. doi: 10.1186/s13052-025-01910-2 (PMC11905720; doi:10.1186/s13052-025-01910-2)

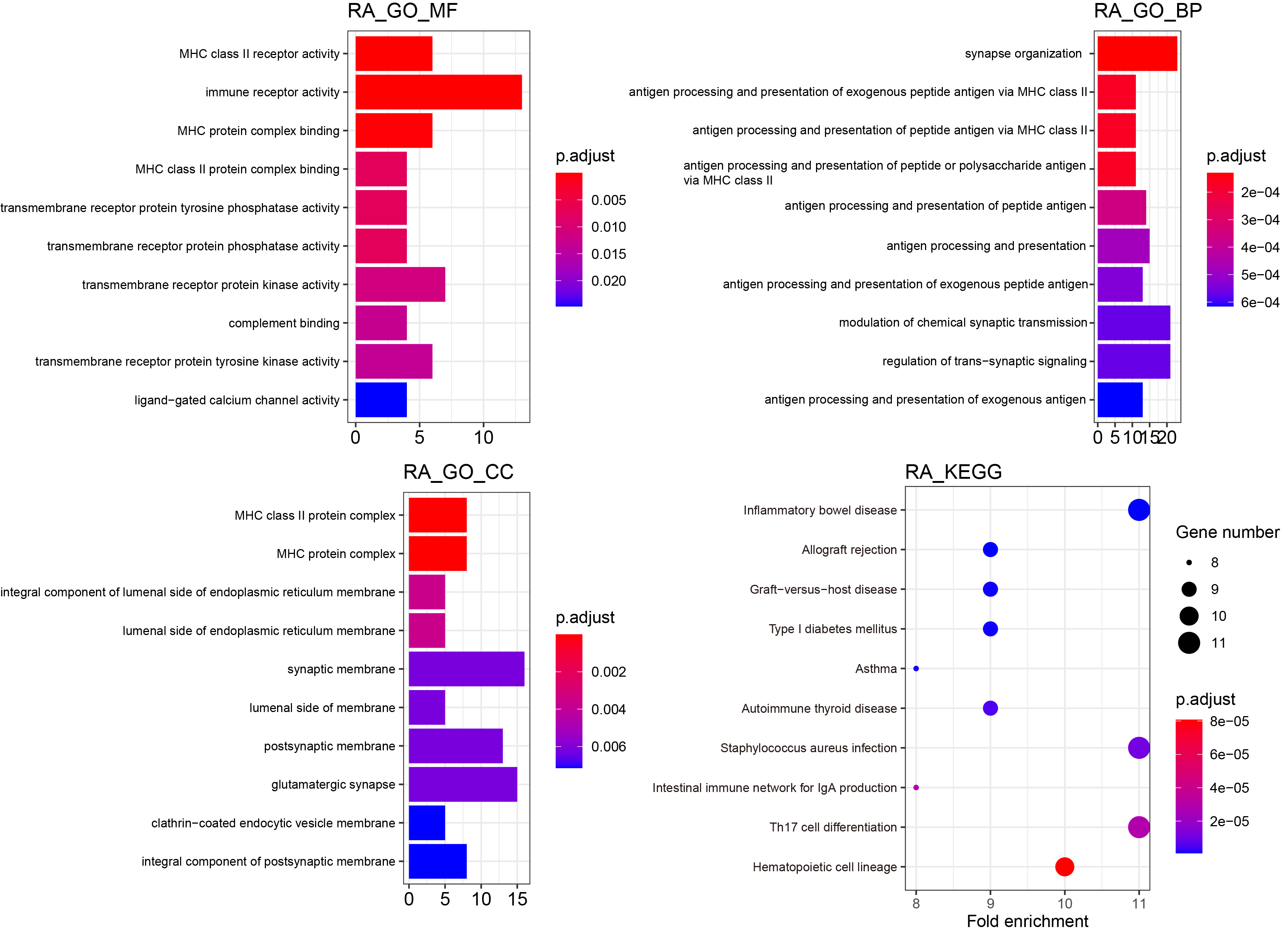

Supplement: Supplementary file 1 — Supplementary Material 1: Supplementary Fig. 1–3. GO and KEGG enrichment analyses of significant SNPs-related genes of SLE, RA and T1D. [file 13052_2025_1910_MOESM1_ESM.zip › Supplementary figure 1 RA.jpg]

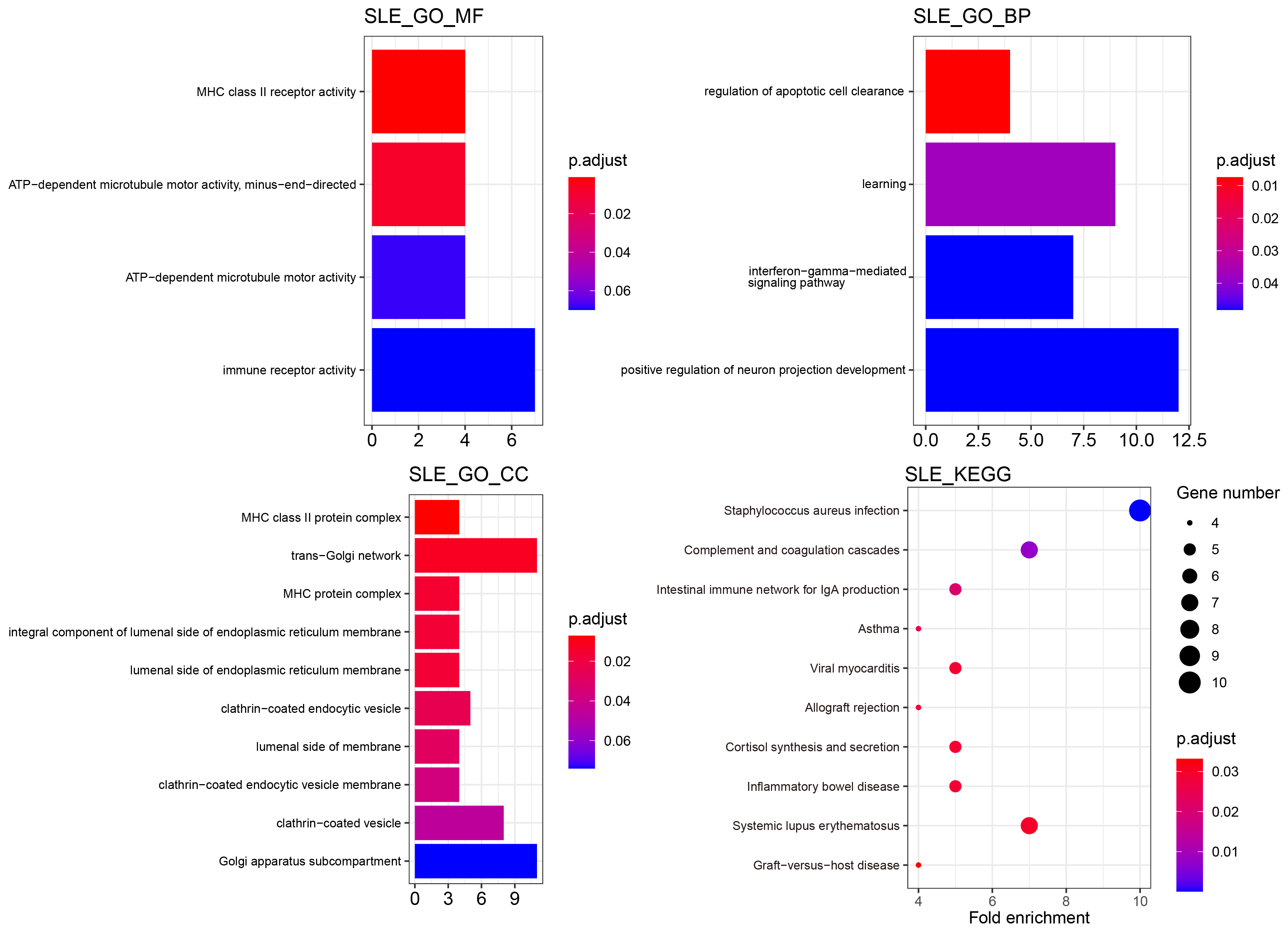

Supplement: Supplementary file 1 — Supplementary Material 1: Supplementary Fig. 1–3. GO and KEGG enrichment analyses of significant SNPs-related genes of SLE, RA and T1D. [file 13052_2025_1910_MOESM1_ESM.zip › Supplementary figure 2 SLE.jpg]

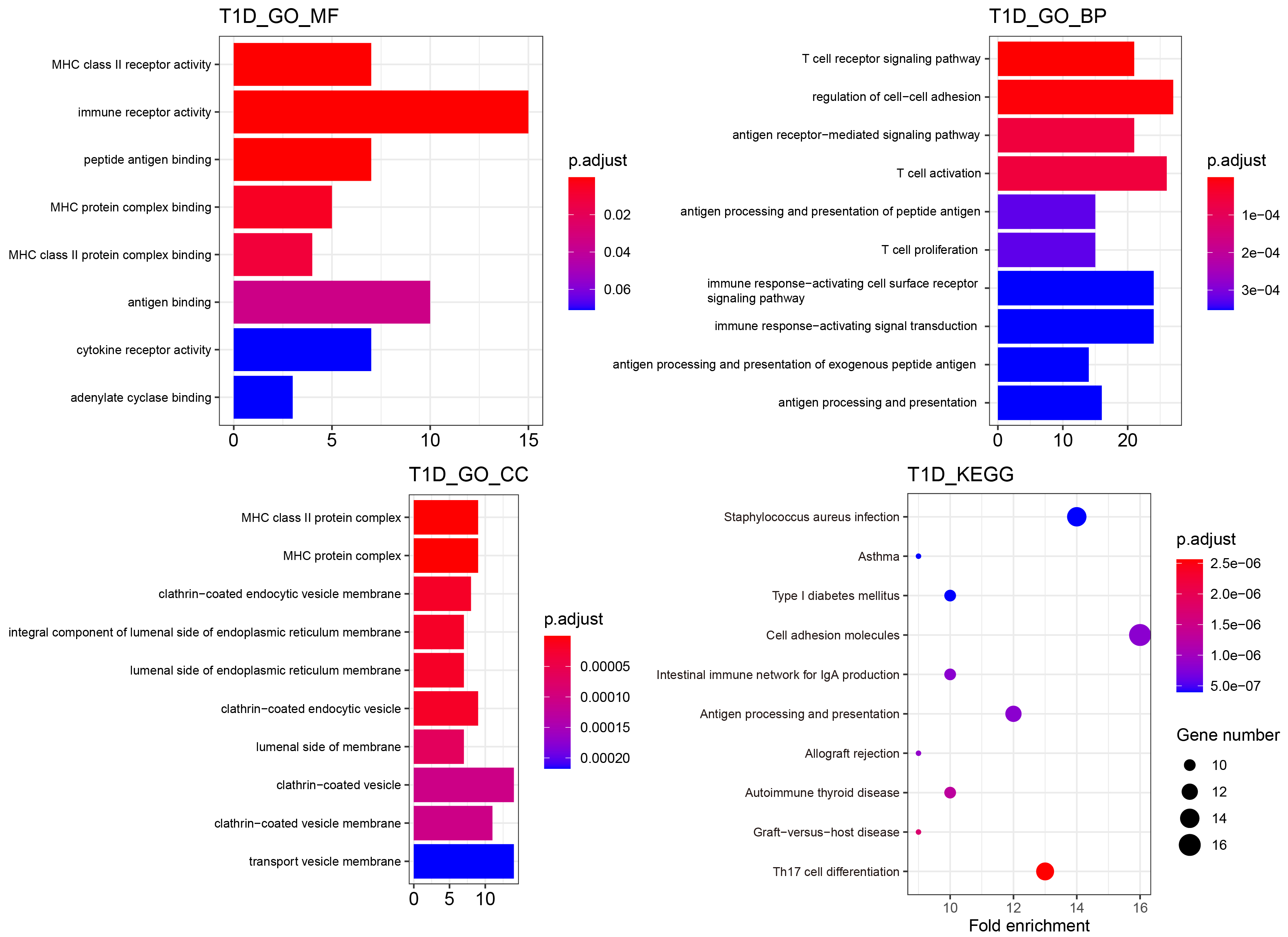

Supplement: Supplementary file 1 — Supplementary Material 1: Supplementary Fig. 1–3. GO and KEGG enrichment analyses of significant SNPs-related genes of SLE, RA and T1D. [file 13052_2025_1910_MOESM1_ESM.zip › Supplementary figure 3 T1D.jpg]
