# Supplementary material for: Association between autoimmune disease and neurodevelopmental disorder: a Mendelian randomization analysis
Source: Ital J Pediatr. 2025 Mar 13;51:76. doi: 10.1186/s13052-025-01910-2 (PMC11905720; doi:10.1186/s13052-025-01910-2)
Supplement: Supplementary file 3 — Supplementary Material 3: Supplementary Table 2. MR results and clincial traits. [file 13052_2025_1910_MOESM3_ESM.docx]

**Supplementary table 2. MR results and clincial traits**

| **MR Analysis** | | | | | | | **Clinical trials** | | |
| --- | --- | --- | --- | --- | --- | --- | --- | --- | --- |
| **Factor’s name** | **GWAS-ID** | **P for MR analysis (IVW)** | **P for pleio-**  **-tropy** | **P for heterogeneity** | | **Number of single SNP** | **Factor’s name** | **Effect size** | **References** |
|  |  |  |  | **MR Egger** | **IVW** |  |  |  |  |
| **ADHD: signal factors as exposures** | | | | | | | **ADHD** | | |
| Granulins | prot-a-1277 | 0.0446 | 0.2845 | 0.3455 | 0.3331 | 25 | Serum Galectin-3 | 1.20±0.78 vs 0.78±0.37 ng/mL; z = -2.303, p = 0.021 ANOVA [F (1,65)=8.731, p=0.004， ηp2=0.118] | Ik, Mit, et al. 2020 |
| Leukocyte immunoglobulin-like receptor subfamily B member 4 | prot-a-1746 | 0.0391 | 0.5143 | 0.201 | 0.2223 | 25 | TNF-α | Attention defict (r =−0.436,p = 0.004); hperactivity (r =−0.376,p = 0.015) | L.-J. Wang, et al. 2022^1^ |
| Semaphorin-3A | prot-a-2669 | 0.0255 | 0.2843 | 0.3373 | 0.3243 | 24 | IL-6 | p=0.03 | Donfrancesco, R. , et al. 2016^2^ |
| Platelet endothelial aggregation receptor 1 | prot-a-2243 | 0.0266 | 0.5293 | 0.8562 | 0.8739 | 24 | IL-10 |  |  |
| Tumor necrosis factor ligand superfamily member 11 | prot-a-3054 | 0.0203 | 0.2069 | 0.3289 | 0.2852 | 20 | IL-6 | 22.35 (95%Cl=(17.68, 26.99)) vs 5.44 (95%Cl=(4.81, 6.06)) | Darwish, A. H. , et al. 2019^3^ |
| Tumor necrosis factor ligand superfamily member 13B | prot-a-3056 | 0.0235 | 0.4675 | 0.1749 | 0.194 | 12 | BDNF | Male: SMD=0.49 (Cl = (0.14,0.84), P=0.006) | J. Zhang, W. Luo, et al. 2017^4^ |
| C-C motif chemokine 20 | prot-a-396 | 0.0278 | 0.5249 | 0.902 | 0.9166 | 16 |  |  |  |
| Ephrin type-A receptor 2 | prot-a-957 | 0.0488 | 0.4041 | 0.9154 | 0.9154 | 16 |  |  |  |
| **ADHD as exposures** | | | | | | |  |  |  |
| Granulins | prot-a-1277 | 0.0273 | 0.8556 | 0.435 | 0.5031 | 18 |  |  |  |
| Platelet glycoprotein V | prot-a-1243 | 0.0406 | 0.708 | 0.401 | 0.46 | 18 |  |  |  |
| Insulin-like growth factor-binding protein 1 | prot-a-1446 | 0.034 | 0.5014 | 0.4781 | 0.5155 | 18 |  |  |  |
| Insulin-like growth factor-binding protein-like 1 | prot-a-1452 | 0.0364 | 0.5618 | 0.9614 | 0.9688 | 18 |  |  |  |
| Interleukin-19 | prot-a-1493 | 0.014 | 0.5652 | 0.8485 | 0.8726 | 18 |  |  |  |
| Interleukin-27 receptor subunit alpha | prot-a-1517 | 0.0266 | 0.5331 | 0.4638 | 0.5061 | 18 |  |  |  |
| Protein jagged-1 | prot-a-1596 | 0.0034 | 0.2278 | 0.9454 | 0.9162 | 18 |  |  |  |
| Leukocyte immunoglobulin-like receptor subfamily B member 2 | prot-a-1743 | 0.0288 | 0.5221 | 0.8664 | 0.8845 | 18 |  |  |  |
| Leukocyte immunoglobulin-like receptor subfamily B member 2 | prot-a-1744 | 0.0325 | 0.6783 | 0.8905 | 0.8091 | 18 |  |  |  |
| Leukocyte immunoglobulin-like receptor subfamily B member 5 | prot-a-1747 | 0.0497 | 0.1463 | 0.8905 | 0.8091 | 18 |  |  |  |
| Platelet endothelial aggregation receptor 1 | prot-a-2243 | 0.0411 | 0.4958 | 0.8191 | 0.839 | 18 |  |  |  |
| Semaphorin-3G | prot-a-2673 | 0.0104 | 0.4533 | 0.6222 | 0.6475 | 18 |  |  |  |
| C-C motif chemokine 3 | prot-a-407 | 0.025 | 0.4174 | 0.1341 | 0.1411 | 18 |  |  |  |
| Pituitary adenylate cyclase-activating polypeptide | prot-a-42 | 0.0404 | 0.8234 | 0.2322 | 0.2845 | 18 |  |  |  |
| Ephrin-A2 | prot-a-897 | 0.0083 | 0.0627 | 0.6516 | 0.435 | 18 |  |  |  |
| Angiopoietin-related protein 1 | prot-a-96 | 0.0264 | 0.5187 | 0.4142 | 0.4532 | 18 |  |  |  |
| **ASD: signal factors as exposures** | | | | | | | **ASD** | | |
| Fibroblast growth factor 10 | prot-a-1087 | 0.007 | 0.649 | 0.776 | 0.825 | 12 | IL-1beta | Hedges’g = 0.652 (CI = (0.304, 0.999), P<0.001 | Masi, A. 2015^5^ |
| Interferon alpha-10 | prot-a-1419 | 0.05 | 0.704 | 0.735 | 0.78 | 20 | IL-6 | Hedges’g = 0.381 (CI = (0.039, 0.722), P=0.03 |  |
| Interleukin-1 receptor antagonist protein | prot-a-1504 | 0.037 | 0.685 | 0.507 | 0.568 | 17 | IL-8 | Hedges’g = 0.455 (CI = (0.024, 0.885), P=0.04 |  |
| Interleukin-22 receptor subunit alpha-2 | prot-a-1511 | 0.044 | 0.223 | 0.724 | 0.681 | 21 | IFN-gamma | Hedges’g = 1.044 (CI = (0.202, 1.885)), P=0.02 |  |
| Interleukin-36 alpha | prot-a-1526 | 0.03 | 0.705 | 0.454 | 0.509 | 21 | eotaxin | Hedges’g = 0.317 (CI = (0.065 to 0.570)), P=0.01 |  |
| Protein jagged-2 | prot-a-1597 | 0.015 | 0.707 | 0.875 | 0.904 | 19 | monocyte chemotactic protein-1/MCP-1 | Hedges’g = 0.257 (CI = (0.005 to 0.508)), P<0.05 |  |
| Leukocyte immunoglobulin-like receptor subfamily B member 1 | prot-a-1742 | 0.037 | 0.144 | 0.879 | 0.805 | 21 | TGF-β1 | Hedges’g = –1.061 (CI = (−1.590 to 0.531)), P<0.001 |  |
| Midkine | prot-a-1871 | 0.012 | 0.266 | 0.704 | 0.68 | 22 | IFN-γ (plasma) | SMD = 0.53 (95%CI=(0.05, 1.00)), P=0.03 | Saghazadeh, A. 2019^6^ |
| Growth/differentiation factor 8 | prot-a-1956 | 0.01 | 0.714 | 0.652 | 0.697 | 27 | IL-1β (serum) | SMD = 0.56(95%CI=(−0.00, 1.13)), P=0.05 |  |
| Tumor necrosis factor receptor superfamily member 16 | prot-a-2043 | 0.028 | 0.998 | 0.999 | 0.999 | 26 | IL-1β | SMD = 0.35 (95%CI=(0.08, 0.61)), P=0.01 |  |
| Prostaglandin reductase 1 | prot-a-2428 | 0.014 | 0.958 | 0.695 | 0.751 | 21 | IL-6 | SMD = 0.33 (95%CI=(0.01, 0.66)), P=0.04 |  |
| Tumor necrosis factor receptor superfamily member 14 | prot-a-3042 | 0.044 | 0.417 | 0.811 | 0.82 | 17 | TNF-α (serum) | SMD = 0.31 (95%CI=(−0.00, 0.61)), P=0.05 |  |
| Tumor necrosis factor receptor superfamily member 1A | prot-a-3046 | 0.04 | 0.545 | 0.635 | 0.668 | 25 | IL-10 (plasma) | SMD = −0.59 (95%CI=(−0.59, −0.04)), P=0.03 | Saghazadeh, A. 2019^7^ |
| Tumor necrosis factor receptor superfamily member 8 | prot-a-3053 | 0.034 | 0.268 | 0.98 | 0.969 | 18 | IL-1Ra (serum) | SMD = −0.25 (95%CI=(−0.46, −0.46)), P=0.01 |  |
| C-C motif chemokine 5 | prot-a-409 | 0.031 | 0.931 | 0.311 | 0.359 | 29 | IL-5 (serum) | SMD = 0.26 (95%CI=(0.00, 0.00)), P=0.05 |  |
| C-X-C motif chemokine 11 | prot-a-739 | 0.011 | 0.815 | 0.554 | 0.623 | 17 | VEGF） | Hedges'g = 0.097 (95%CI = (0.018, 0.175)), P = 0.016 | Liu, S. H. 2021^8^ |
| Ephrin type-A receptor 5 | prot-a-958 | 0.048 | 0.62 | 0.632 | 0.682 | 17 |  |  |  |
| Erythropoietin | prot-a-967 | 0.048 | 0.36 | 0.852 | 0.846 | 16 |  |  |  |
| **ASD as exposures** | | | | | | |  |  |  |
| TNF superfamily member 10 | prot-b-27 | 0.003 | 0.499 | 0.295 | 0.333 | 21 |  |  |  |
| platelet derived growth factor subunit B | prot-b-13 | 0.014 | 0.77 | 0.608 | 0.69 | 21 |  |  |  |
| Fibroblast growth factor 16 | prot-a-1089 | 0.018 | 0.997 | 0.418 | 0.482 | 21 |  |  |  |
| Semaphorin-3G | prot-a-2673 | 0.018 | 0.964 | 0.388 | 0.451 | 21 |  |  |  |
| Angiopoietin-related protein 1 | prot-a-96 | 0.02 | 0.412 | 0.18 | 0.189 | 21 |  |  |  |
| vascular endothelial growth factor A | prot-b-22 | 0.025 | 0.794 | 0.196 | 0.259 | 21 |  |  |  |
| C-C motif chemokine 28 | prot-a-406 | 0.027 | 0.073 | 0.661 | 0.486 | 21 |  |  |  |
| Tumor necrosis factor receptor superfamily member 14 | prot-a-3042 | 0.028 | 0.797 | 0.435 | 0.495 | 21 |  |  |  |
| **Schizophrenia: signal factors as exposures** | | | | | | | **Schizophrenia** | | |
| Tumor necrosis factor receptor superfamily member 6 | prot-a-1057 | 0.029 | 0.317 | 0.194 | 0.184 | 16 | IL-6 | Cohen’s d = 0.339 (CI= (0.192 - 0.487)), P<0.001 | Fraguas, D. 2019^9^ |
| Fibroblast growth factor 16 | prot-a-1090 | 0.043 | 0.601 | 0.294 | 0.339 | 16 | TNF-α | Cohen’s d = 0.432 (CI= (0.157 - 0.708)), P<0.001 |  |
| Fibroblast growth factor 5 | prot-a-1095 | 0.03 | 0.819 | 0.093 | 0.117 | 25 | IL-6 (serum) | SMD = 0.44 (95%CI = (0.34 - 0.55)), P<0.00001 | Zhou, X. 2021^10^ |
| Fibroblast growth factor receptor 4 | prot-a-1105 | 0.025 | 0.389 | 0.208 | 0.215 | 24 | IL-1β | Hedge'sg ± SE = 1.174 ± 0.129, P<0.001 | Upthegrove, R. 2014^11^ |
| Interferon alpha-8 | prot-a-1425 | 0.019 | 0.695 | 0.33 | 0.39 | 15 | IL-6 | Hedge'sg ± SE = 2.206 ± 0.890, P=0.013 |  |
| Interferon gamma | prot-a-1429 | 0.015 | 0.328 | 0.488 | 0.488 | 23 | TNF-α | Hedge'sg ± SE = 0.944 ± 0.126, P<0.00001 |  |
| Interleukin-1 beta | prot-a-1495 | 0.034 | 0.278 | 0.516 | 0.493 | 15 | IL-6 (cerebrospinal fluid) | SMD = 0.53 (95%CI = (0.28 - 0.78)), P<0.001 | Gallego, J. 2018^12^ |
| Interleukin-1 receptor type 2 | prot-a-1498 | 0.016 | 0.389 | 0.618 | 0.629 | 13 | IL-8(cerebrospinal fluid) | SMD = 1.12 (95%CI = (0.16-2.09)), P=0.02 |  |
| Interleukin-27 receptor subunit alpha | prot-a-1517 | 0.001 | 0.074 | 0.941 | 0.815 | 19 | IL-6 (serum) | β= 3.60 (CI = (1.35 - 5.86))，P = 0.002 | Challa, F. 2021^13^ |
| Interleukin-4 | prot-a-1532 | 0.036 | 0.608 | 0.403 | 0.45 | 20 | TNF-α | Case: Mean ± SEM = 27.04 ± 4.11 pg/mL  Control: Mean ± SEM = 17.25 ± 3.3 pg/mL  P<0.01 | Al-Asmari, A. K. 2014^14^ |
| LIM and cysteine-rich domains protein 1 | prot-a-1757 | 0.032 | 0.678 | 0.703 | 0.757 | 15 | IL-6 | Case: Mean ± SEM = 81.58 ± 18.06 pg/mL  Control: Mean ± SEM = 62.12 ± 10.08 pg/mL  P＜0.05 |  |
| Prostaglandin-H2 D-isomerase | prot-a-2426 | 0.007 | 0.222 | 0.063 | 0.051 | 31 | IL-1β | Case: Mean ± SEM = 81.58 ± 18.06 pg/mL  Control: Mean ± SEM = 16.62 ± 4.6 pg/mL  P＜0.01 |  |
| Tumor necrosis factor receptor superfamily member 19L | prot-a-2520 | 0.007 | 0.759 | 0.135 | 0.176 | 14 | IFN-γ | Case: Mean ± SEM = 24.52 ± 3.97 pg/mL  Control: Mean ± SEM = 52.53 ± 8.03 pg/mL  P＜0.05 |  |
| Semaphorin-3C | prot-a-2671 | 0.017 | 0.244 | 0.929 | 0.889 | 14 | CCL17 | Case: Median = 89.62 (IQR = (66.15–125.82) pg/mL  Control: Median = 48.60 (IQR = (28.03–71.71)) pg/mL  P=0.001 | Malmqvist, A. 2019^15^ |
| Transforming growth factor beta-1-induced transcript 1 protein | prot-a-2963 | 0.028 | 0.484 | 0.498 | 0.534 | 17 | IL-6 | Case: Median = 5.31 (IQR = (0.85-17.20) ng/mL  Control: Median = 2.42 (IQR = (0.54-9.36)) ng/mL  P=0.02 | Miller, B. 2021^16^ |
| Thrombopoietin | prot-a-2972 | 0.016 | 0.384 | 0.818 | 0.821 | 21 | BAFF | Case: Mean ± SD = 743.66 ± 253.39 pg/mL  Control: Mean ± SD = 1037.14 ± 339.74 pg/mL  P<0.001 | El Kissi, Y. 2015^17^ |
| Tumor necrosis factor ligand superfamily member 4 | prot-a-3062 | 0.03 | 0.912 | 0.721 | 0.776 | 20 | IL-17 | Case: Median = 110.00 (IQR = (52.50 – 215.00)) pg/mL  Control: Median = 40 (IQR = (0.00 – 53.75)) pg/mL  P<0.001 |  |
| Eotaxin | prot-a-387 | 0.036 | 0.912 | 0.353 | 0.404 | 29 | MCP-1 (plasma) | Cohen’s d = 0.82, P=0.0015 | Orhan, F. 2018^18^ |
| C-C motif chemokine 8 | prot-a-411 | 0.034 | 0.801 | 0.874 | 0.904 | 21 | IL-18 (serum) | F = 31.362, df = 1, 143, P < 0.001 | Wu, J. 2018^19^ |
| Cysteine-rich hydrophobic domain-containing protein 2 | prot-a-542 | 0.013 | 0.793 | 0.784 | 0.823 | 25 | IL-17 (plasma) | Case: Mean ± SD = 37.63±17.82 pg/mL  Control: Mean ± SD = 29.34±10.38 pg/mL  P=0.02 | Li, H. 2016^20^ |
| Cysteine-rich secretory protein 2 | prot-a-661 | 0.045 | 0.532 | 0.321 | 0.352 | 25 | IL-23 (plasma) | Case: Mean ± SD = 101.40±135.26 pg/mL  Control: Mean ± SD = 13.09±5.94 pg/mL  P=0.01 |  |
| Interleukin-8 | prot-a-749 | 0.014 | 0.14 | 0.653 | 0.551 | 19 | TGF-β1 (plasma) | Case: Mean ± SD = 2864.57±2163.61 pg/mL  Control: Mean ± SD = 1839.69±1797.73 pg/mL  P=0.04 |  |
| Ephrin-B2 | prot-a-905 | 0.031 | 0.637 | 0.686 | 0.736 | 16 | IL-3 (serum) | F=16.35， df=1，82， p<0.001 | Xiu, M. 2015^21^ |
| TNF superfamily member 14 | prot-b-24 | 0.026 | 0.436 | 0.22 | 0.232 | 5 | TNF-α (plasma) | Cohen’s d = 0.57, P<0.001 | Ellen E. 2017 |
| nerve growth factor | prot-b-40 | 0.013 | 0.93 | 0.448 | 0.576 | 7 | IL-6 (plasma) | Cohen’s d = 0.53, P<0.001 |  |
| **Schizophrenia: signal factors as exposures** | | | | | | |  |  |  |
| Interleukin-5 | prot-a-1535 | 0.042 | 0.844 | 0.855 | 0.889 | 22 |  |  |  |
| Netrin-1 | prot-a-2117 | 0.01 | 0.278 | 0.808 | 0.788 | 22 |  |  |  |
| Semaphorin-3A | prot-a-2669 | 0.008 | 0.494 | 0.348 | 0.377 | 22 |  |  |  |
| Angiopoietin-like protein 8 | prot-a-292 | 0.025 | 0.768 | 0.69 | 0.74 | 22 |  |  |  |
| Tumor necrosis factor receptor superfamily member 12A | prot-a-3040 | 0.02 | 0.061 | 0.777 | 0.588 | 22 |  |  |  |
| Vascular endothelial growth factor C | prot-a-3199 | 0.029 | 0.107 | 0.615 | 0.494 | 22 |  |  |  |
| Tumor necrosis factor ligand superfamily member 4 | prot-a-3061 | 0.033 | 0.711 | 0.915 | 0.935 | 22 |  |  |  |
| Stem Cell Growth Factor-beta | prot-a-569 | 0.028 | 0.983 | 0.551 | 0.614 | 22 |  |  |  |
| Cysteine-rich secretory protein 2 | prot-a-661 | 0.013 | 0.087 | 0.451 | 0.325 | 22 |  |  |  |
| Angiopoietin-1 | prot-a-92 | 0.048 | 0.29 | 0.825 | 0.808 | 22 |  |  |  |
| TNF superfamily member 14 | prot-b-24 | 0.04 | 0.553 | 0.224 | 0.264 | 11 |  |  |  |
| resistin | prot-b-32 | 0.034 | 0.753 | 0.486 | 0.571 | 11 |  |  |  |
| growth differentiation factor 15 | prot-b-55 | 0.026 | 0.249 | 0.477 | 0.432 | 11 |  |  |  |
|  | | | | | | | | | |
| **SLE: signal mediators as exposures** | | | | | | | **SLE** | | |
| Interferon gamma receptor 2 | prot-a-1433 | 0.021 | 0.542 | 0.77 | 0.809 | 10 | adiponectin | SMD = 0.502 (95% CI = (0.021-0.984)), P < 0.001 | Dini, A. A. 2017^22^ |
| Insulin-like growth factor-binding protein 1 | prot-a-1446 | 0.009 | 0.147 | 0.952 | 0.87 | 14 | IL-17 | SMD = 1.183 (95% CI = (0.763, 1.603), P < 0.001 | Shen, H. H. 2020^23^ |
| Platelet-derived growth factor receptor alpha | prot-a-2229 | 0.037 | 0.924 | 0.767 | 0.82 | 18 | HGF | P < 0.005 | Robak, E. 2001^24^ |
| Tumor necrosis factor receptor superfamily member 14 | prot-a-3042 | 0.014 | 0.618 | 0.746 | 0.788 | 17 | IL-8 | SMD = 0.963 (95% CI = (0.416, 1.511)) | Mao, Y. M. 2018^25^ |
| Tumor necrosis factor ligand superfamily member 9 | prot-a-3064 | 0.048 | 0.609 | 0.117 | 0.141 | 17 | resistin |  | Huang, Q. |
| Gro-beta/gamma | prot-a-747 | 0.005 | 0.102 | 0.815 | 0.679 | 19 | MIF | SMD = 1.154 (95% CI = (0.369, 1.938)), P = 0.004 | Bae, S. C. 2017^26^ |
| Epidermal growth factor receptor variant III | prot-a-910 | 0.01 | 0.906 | 0.552 | 0.632 | 14 | FGF-23 | 106.7 (80.3-179) vs. 33.6 (25.8-60.9) pg/ml, p<0.001 | Resende, A. L. 2017^27^ |
| TNF superfamily member 11 | prot-b-12 | 0.036 | 0.609 | 0.238 | 0.352 | 3 | IFN-α |  | Denny, M. F.2007^28^ |
| **SLE: signal mediators as exposures** | | | | | | | IL-6 |  | Ding, J., et al. 2020.^29^ |
| Fibroblast growth factor 6 | prot-a-1096 | 0.013 | 0.841 | 0.742 | 0.788 | 24 | IL-10 |  | Llorente, L.2000^30^ |
| Interferon alpha-6 | prot-a-1423 | 0.03 | 0.713 | 0.46 | 0.512 | 24 | BMP-2 |  | Tang, Y.2013^31^ |
| Insulin-like growth factor-binding protein 1 | prot-a-1477 | 0.041 | 0.129 | 0.65 | 0.556 | 24 | Kininogen |  | Dellalibera-Joviliano, R.2001^32^ |
| Interleukin-31 | prot-a-1521 | 0.041 | 0.915 | 0.097 | 0.123 | 24 | leptin |  | Li, H. M.2015^33^ |
| Interferon regulatory factor 1 | prot-a-1567 | 0.049 | 0.802 | 0.161 | 0.196 | 24 | IL-18 |  | Xiang, M., et al. 2021. ^34^ |
| Oncostatin-M-specific receptor subunit beta | prot-a-2156 | 0.002 | 0.184 | 0.78 | 0.726 | 24 | TNFsf14 |  | González-Serna, D.2018.^35^ |
| Prostaglandin-H2 D-isomerase | prot-a-2426 | 0.044 | 0.389 | 0.119 | 0.122 | 24 | MMP |  | Lee, J. M.2019.^36^ |
| Ephrin-A3 | prot-a-899 | 0.045 | 0.28 | 0.66 | 0.642 | 24 | VEGF |  | Robak, E.2001.^24^ |
| Epidermal growth factor receptor variant III | prot-a-910 | 0.044 | 0.724 | 0.852 | 0.881 | 24 |  |  |  |
| Ephrin type-B receptor 2 | prot-a-960 | 0.028 | 0.015 | 0.877 | 0.542 | 24 |  |  |  |
| **RA: signal mediators as exposures** | | | | | | | **RA** | | |
| Fibroblast growth factor 16 | prot-a-1089 | 0.036 | 0.986 | 0.1 | 0.131 | 19 | Circulating leptin level | SMD = 1.056（95 % CI = 0.647, 1.465）, P = 4.2 × 10-7 | Y H Lee. 2016^37^ |
| 15-hydroxyprostaglandin dehydrogenase [NAD(+)] | prot-a-1370 | 0.01 | 0.853 | 0.239 | 0.284 | 25 | Insulin-like growth factor-1 (IGF-1) | SMD = -0.936，(95% CI = -1.382, -0.489), P<0.001 | Yu-Lan Zhao. 2019^38^ |
| Interleukin-16 | prot-a-1479 | 0.004 | 0.818 | 0.851 | 0.882 | 25 | TNFα | P=0.019 | Ilaria Buondonno. 2017^39^ |
| Interleukin-17A | prot-a-1481 | 0.019 | 0.664 | 0.061 | 0.077 | 17 | TGFβ | P=0.006 |  |
| Interleukin-15 receptor subunit alpha | prot-a-1478 | 0.022 | 0.873 | 0.568 | 0.632 | 20 | IL-23 | P=0.018 |  |
| Leukocyte immunoglobulin-like receptor subfamily A member 5 | prot-a-1739 | 0.025 | 0.888 | 0.435 | 0.506 | 17 | IL-6 | P=0.0004 |  |
| Tumor necrosis factor receptor superfamily member 11B | prot-a-3039 | 0.022 | 0.886 | 0.26 | 0.318 | 18 | CXCL-5(ENA-78) | RA (70 +/- 26 ng/ml)；  Normal (0.12 +/- 0.04 ng/ml)  P < 0.05 | A E Koch. 1994^40^ |
| Tumor necrosis factor | prot-a-3029 | 0.05 | 0.118 | 0.175 | 0.089 | 14 | IL-20 | P < 0.002 | Kragstrup, T. W. 2016^41^ |
| C-C motif chemokine 20 | prot-a-396 | 0.041 | 0.287 | 0.629 | 0.607 | 16 | IL-24 | P < 0.002 |  |
| Tumor necrosis factor receptor superfamily member 1A | prot-a-3046 | 0.045 | 0.846 | 0.273 | 0.321 | 25 | IL-12 | RA median(range)=2.7 (0, 170.8) pg/ml; Health median(range)=0 (0, 10.9) pg/ml,  P < 0.001 | W -U Kim. 2000^42^ |
| Tumor necrosis factor ligand superfamily member 14 | prot-a-3057 | 0.05 | 0.902 | 0.679 | 0.75 | 14 |  |  |  |
| Cysteine and glycine-rich protein 3 | prot-a-701 | 0.026 | 0.295 | 0.61 | 0.589 | 14 |  |  |  |
| Angiopoietin-4 | prot-a-95 | 0.029 | 0.593 | 0.272 | 0.305 | 27 |  |  |  |
| **RA as exposures** | | | | | | |  |  |  |
| Heparin-binding EGF-like growth factor | prot-a-1313 | 0.004 | 0.34 | 0.814 | 0.813 | 44 |  |  |  |
| Granulins | prot-a-1277 | 0.044 | 0.542 | 0.332 | 0.356 | 44 |  |  |  |
| Growth/differentiation factor 11/8 | prot-a-1194 | 0.046 | 0.864 | 0.928 | 0.942 | 44 |  |  |  |
| Interferon lambda-1 | prot-a-1435 | 0.033 | 0.798 | 0.506 | 0.546 | 44 |  |  |  |
| Interferon-induced protein with tetratricopeptide repeats 2 | prot-a-1416 | 0.044 | 0.061 | 0.3 | 0.205 | 44 |  |  |  |
| Insulin-like growth factor-binding protein 3 | prot-a-1449 | 0.049 | 0.443 | 0.748 | 0.76 | 44 |  |  |  |
| Interleukin-17C | prot-a-1483 | 0.013 | 0.197 | 0.75 | 0.719 | 44 |  |  |  |
| Interleukin-18-binding protein | prot-a-1490 | 0.032 | 0.465 | 0.762 | 0.776 | 44 |  |  |  |
| Interleukin-17 receptor B | prot-a-1487 | 0.041 | 0.952 | 0.705 | 0.742 | 44 |  |  |  |
| Interleukin-13 receptor subunit alpha-1 | prot-a-1476 | 0.046 | 0.216 | 0.859 | 0.838 | 44 |  |  |  |
| Interleukin enhancer-binding factor 3 | prot-a-1547 | 0.014 | 0.961 | 0.416 | 0.459 | 44 |  |  |  |
| Midkine | prot-a-1871 | 0.042 | 0.744 | 0.949 | 0.958 | 44 |  |  |  |
| Interferon regulatory factor 2 | prot-a-1568 | 0.043 | 0.632 | 0.508 | 0.541 | 44 |  |  |  |
| Bone morphogenetic protein receptor type-1A | prot-a-259 | 0.021 | 0.778 | 0.86 | 0.882 | 44 |  |  |  |
| Platelet receptor Gi24 | prot-a-281 | 0.043 | 0.197 | 0.502 | 0.471 | 44 |  |  |  |
| Tumor necrosis factor | prot-a-3029 | 0.049 | 0.44 | 0.089 | 0.094 | 44 |  |  |  |
| C-C motif chemokine 22 | prot-a-398 | 0.011 | 0.226 | 0.075 | 0.065 | 44 |  |  |  |
| C-C motif chemokine 17 | prot-a-394 | 0.015 | 0.477 | 0.603 | 0.623 | 44 |  |  |  |
| C-C motif chemokine 14 | prot-a-390 | 0.034 | 0.533 | 0.657 | 0.681 | 44 |  |  |  |
| C-C motif chemokine 14 | prot-a-390 | 0.034 | 0.533 | 0.657 | 0.681 | 44 |  |  |  |
| Gro-beta/gamma | prot-a-746 | 0.006 | 0.473 | 0.244 | 0.259 | 44 |  |  |  |
| Scavenger receptor cysteine-rich type 1 protein M130 | prot-a-419 | 0.016 | 0.977 | 0.211 | 0.243 | 44 |  |  |  |
| Ephrin-A4 | prot-a-900 | 0.045 | 0.979 | 0.776 | 0.808 | 44 |  |  |  |
| C-X-C motif chemokine 5 | prot-a-748 | 0.047 | 0.925 | 0.078 | 0.095 | 44 |  |  |  |
| resistin | prot-b-32 | 0.002 | 0.998 | 0.422 | 0.494 | 44 |  |  |  |
| nerve growth factor | prot-b-40 | 0.015 | 0.293 | 0.354 | 0.34 | 44 |  |  |  |
| **T1D: signal mediators as exposures** | | | | | | | **T1D** | | |
| Fibroblast growth factor 16 | prot-a-1089 | 0.034 | 0.797 | 0.748 | 0.798 | 19 | TNF-α | SMD=1.23 (Cl = (0.77, 1.68)), P<0.001 | Qiao, Y. C., et al. (2017)^43^ |
| Glia maturation factor gamma | prot-a-1225 | 0.049 | 0.054 | 0.478 | 0.312 | 24 | CD4+CD25+Treg | SMD=-1.64 (Cl = (-2.52, -0.75)), P=0.00003 | Qiao, Y. C., et al. (2016)^44^ |
| Interferon gamma | prot-a-1429 | 0.041 | 0.294 | 0.868 | 0.854 | 23 | CD4+CD25+Foxp3+Treg, | SMD=-1.38 (Cl = (-2.52, -0.24)), P=0.020 |  |
| Interferon alpha-8 | prot-a-1425 | 0.048 | 0.199 | 0.643 | 0.57 | 15 | TGF-β | SMD=-1.97(Cl = (-3.79, -0.15)), P=0.030 |  |
| Interleukin-17C | prot-a-1483 | 0.011 | 0.385 | 0.437 | 0.453 | 15 | IL-6 | SMD=1.49 (Cl = (1.04, 1.93)), P<0.001 |  |
| Interleukin-12 | prot-a-1469 | 0.022 | 0.697 | 0.277 | 0.332 | 15 | IFN-γ | 14.85±1.31 vs 16.36±1.24, p=0.001 | Vaseghi, H., et al. (2016)^45^ |
| Interleukin-11 receptor subunit alpha | prot-a-1467 | 0.049 | 0.407 | 0.471 | 0.494 | 12 | IL-4 | 19.08±2.42 vs 15.79±1.71, p<0.001 |  |
| Interleukin-27 receptor subunit alpha | prot-a-1517 | 0 | 0.568 | 0.389 | 0.433 | 19 |  |  |  |
| Interleukin-37 | prot-a-1529 | 0.015 | 0.987 | 0.827 | 0.864 | 25 |  |  |  |
| Interleukin-22 receptor subunit alpha-2 | prot-a-1511 | 0.025 | 0.535 | 0.475 | 0.515 | 21 |  |  |  |
| Interleukin-36 gamma | prot-a-1528 | 0.045 | 0.904 | 0.659 | 0.715 | 23 |  |  |  |
| Protein jagged-1 | prot-a-1596 | 0.024 | 0.623 | 0.843 | 0.868 | 25 |  |  |  |
| Interleukin enhancer-binding factor 3 | prot-a-1547 | 0.037 | 0.23 | 0.935 | 0.892 | 14 |  |  |  |
| Growth/differentiation factor 8 | prot-a-1957 | 0.04 | 0.189 | 0.865 | 0.798 | 16 |  |  |  |
| Leukocyte immunoglobulin-like receptor subfamily A member 5 | prot-a-1739 | 0.04 | 0.131 | 0.951 | 0.877 | 17 |  |  |  |
| Tumor necrosis factor receptor superfamily member 19L | prot-a-2519 | 0.02 | 0.631 | 0.527 | 0.586 | 15 |  |  |  |
| Bone morphogenetic protein 10 | prot-a-254 | 0.02 | 0.931 | 0.202 | 0.264 | 13 |  |  |  |
| Platelet endothelial aggregation receptor 1 | prot-a-2244 | 0.042 | 0.481 | 0.779 | 0.799 | 19 |  |  |  |
| Transforming growth factor beta-1-induced transcript 1 protein | prot-a-2963 | 0.018 | 0.965 | 0.523 | 0.596 | 17 |  |  |  |
| Tumor necrosis factor receptor superfamily member 11B | prot-a-3039 | 0.019 | 0.254 | 0.613 | 0.581 | 18 |  |  |  |
| Complement C1q tumor necrosis factor-related protein 3 | prot-a-304 | 0.021 | 0.425 | 0.826 | 0.833 | 11 |  |  |  |
| Transforming growth factor beta-2 | prot-a-2964 | 0.042 | 0.266 | 0.298 | 0.277 | 20 |  |  |  |
| C-C motif chemokine 25 | prot-a-402 | 0.012 | 0.09 | 0.62 | 0.457 | 18 |  |  |  |
| C-X-C motif chemokine 13 | prot-a-743 | 0.017 | 0.131 | 0.79 | 0.693 | 21 |  |  |  |
| Gro-beta/gamma | prot-a-746 | 0.035 | 0.907 | 0.189 | 0.228 | 26 |  |  |  |
| Connective tissue growth factor | prot-a-712 | 0.042 | 0.045 | 0.337 | 0.154 | 19 |  |  |  |
| Desert hedgehog protein N-product | prot-a-813 | 0.024 | 0.712 | 0.458 | 0.529 | 14 |  |  |  |
| Ephrin type-B receptor 2 | prot-a-961 | 0.028 | 0.882 | 0.691 | 0.742 | 24 |  |  |  |
| Interleukin-8 | prot-a-749 | 0.039 | 0.812 | 0.305 | 0.363 | 190 |  |  |  |
| vascular endothelial growth factor D | prot-b-65 | 0.006 | 1 | 0.647 | 0.746 | 9 |  |  |  |
| **T1D as exposures** | | | | | | |  |  |  |
| Growth/differentiation factor 11 | prot-a-1193 | 0.038 | 0.705 | 0.805 | 0.822 | 95 |  |  |  |
| Fibroblast growth factor 7 | prot-a-1097 | 0.005 | 0.715 | 0.626 | 0.65 | 95 |  |  |  |
| Fibroblast growth factor 9 | prot-a-1101 | 0.01 | 0.717 | 0.306 | 0.328 | 95 |  |  |  |
| Insulin-like growth factor-binding protein 7 | prot-a-1451 | 0.001 | 0.771 | 0.305 | 0.328 | 95 |  |  |  |
| Interferon alpha/beta receptor 1 | prot-a-1426 | 0.009 | 0.519 | 0.215 | 0.227 | 95 |  |  |  |
| Interferon gamma | prot-a-1428 | 0.01 | 0.717 | 0.306 | 0.328 | 95 |  |  |  |
| Interferon alpha-6 | prot-a-1423 | 0.02 | 0.983 | 0.456 | 0.485 | 95 |  |  |  |
| Interleukin-1 receptor type 2 | prot-a-1498 | 0.025 | 0.168 | 0.774 | 0.75 | 95 |  |  |  |
| Interleukin-12 receptor subunit beta-1 | prot-a-1473 | 0.007 | 0.967 | 0.49 | 0.519 | 95 |  |  |  |
| Interleukin-12 | prot-a-1468 | 0.038 | 0.574 | 0.158 | 0.169 | 95 |  |  |  |
| Interleukin-22 receptor subunit alpha-2 | prot-a-1510 | 0.002 | 0.862 | 0.163 | 0.18 | 95 |  |  |  |
| Interleukin-22 receptor subunit alpha-1 | prot-a-1509 | 0.025 | 0.168 | 0.774 | 0.75 | 95 |  |  |  |
| Interleukin-7 | prot-a-1543 | 0.042 | 0.492 | 0.66 | 0.674 | 95 |  |  |  |
| Agouti-signaling protein | prot-a-187 | 0 | 0.401 | 0.817 | 0.822 | 95 |  |  |  |
| Interferon regulatory factor 4 | prot-a-1569 | 0 | 0.629 | 0.517 | 0.54 | 95 |  |  |  |
| Leukocyte immunoglobulin-like receptor subfamily A member 5 | prot-a-1740 | 0.002 | 0.799 | 0.664 | 0.689 | 95 |  |  |  |
| Leukotriene A-4 hydrolase | prot-a-1804 | 0.012 | 0.335 | 0.177 | 0.177 | 95 |  |  |  |
| Interferon regulatory factor 2 | prot-a-1568 | 0.042 | 0.639 | 0.942 | 0.948 | 95 |  |  |  |
| Platelet endothelial aggregation receptor 1 | prot-a-2244 | 0 | 0.401 | 0.817 | 0.822 | 95 |  |  |  |
| Platelet factor 4 variant | prot-a-2251 | 0.002 | 0.741 | 0.248 | 0.269 | 95 |  |  |  |
| Oncostatin-M | prot-a-2154 | 0.001 | 0.892 | 0.086 | 0.098 | 95 |  |  |  |
| Bone morphogenetic protein receptor type-1A | prot-a-259 | 0.021 | 0.135 | 0.522 | 0.485 | 95 |  |  |  |
| Parathyroid hormone-related protein | prot-a-2432 | 0.036 | 0.545 | 0.639 | 0.656 | 95 |  |  |  |
| Platelet-derived growth factor subunit B | prot-a-2226 | 0.042 | 0.639 | 0.942 | 0.948 | 95 |  |  |  |
| Tumor necrosis factor receptor superfamily member 19L | prot-a-2519 | 0.042 | 0.147 | 0.186 | 0.163 | 95 |  |  |  |
| Placenta growth factor | prot-a-2254 | 0.048 | 0.075 | 0.515 | 0.45 | 95 |  |  |  |
| Tumor necrosis factor alpha-induced protein 3 | prot-a-3030 | 0.001 | 0.714 | 0.404 | 0.429 | 95 |  |  |  |
| Tumor necrosis factor receptor superfamily member 18 | prot-a-3044 | 0.001 | 0.892 | 0.086 | 0.098 | 95 |  |  |  |
| Tumor necrosis factor | prot-a-3029 | 0.004 | 0.343 | 0.065 | 0.065 | 95 |  |  |  |
| Tumor necrosis factor receptor superfamily member 11A | prot-a-3037 | 0.042 | 0.386 | 0.419 | 0.425 | 95 |  |  |  |
| Tumor necrosis factor receptor superfamily member 17 | prot-a-3043 | 0.012 | 0.178 | 0.177 | 0.16 | 95 |  |  |  |
| Semaphorin-6A | prot-a-2677 | 0.023 | 0.956 | 0.163 | 0.181 | 95 |  |  |  |
| C-C motif chemokine 21 | prot-a-397 | 0 | 0.011 | 0.125 | 0.056 | 95 |  |  |  |
| Tumor necrosis factor receptor superfamily member 4 | prot-a-3051 | 0.049 | 0.963 | 0.295 | 0.321 | 95 |  |  |  |
| Vascular endothelial growth factor C | prot-a-3199 | 0.008 | 0.558 | 0.611 | 0.629 | 95 |  |  |  |
| Tumor necrosis factor ligand superfamily member 12 | prot-a-3055 | 0.009 | 0.909 | 0.977 | 0.981 | 95 |  |  |  |
| C-C motif chemokine 22 | prot-a-398 | 0.01 | 0.593 | 0.13 | 0.14 | 95 |  |  |  |
| Tumor necrosis factor ligand superfamily member 15 | prot-a-3059 | 0.027 | 0.09 | 0.739 | 0.687 | 95 |  |  |  |
| C-C motif chemokine 14 | prot-a-390 | 0.046 | 0.428 | 0.15 | 0.156 | 95 |  |  |  |
| C-X-C motif chemokine 14 | prot-a-744 | 0.021 | 0.51 | 0.279 | 0.293 | 95 |  |  |  |
| Ciliary neurotrophic factor | prot-a-604 | 0.01 | 0.837 | 0.839 | 0.856 | 95 |  |  |  |
| Cysteine-rich motor neuron 1 protein | prot-a-660 | 0.01 | 0.266 | 0.99 | 0.989 | 95 |  |  |  |
| C-C motif chemokine 3 | prot-a-407 | 0.015 | 0.048 | 0.01 | 0.005 | 95 |  |  |  |
| C-C motif chemokine 3-like 1 | prot-a-408 | 0.031 | 0.117 | 0.038 | 0.028 | 95 |  |  |  |
| Delta and Notch-like epidermal growth factor-related receptor | prot-a-853 | 0 | 0.952 | 0.448 | 0.477 | 95 |  |  |  |
| Ephrin type-A receptor 1 | prot-a-956 | 0.019 | 0.728 | 0.432 | 0.457 | 95 |  |  |  |
| Ephrin-B2 | prot-a-904 | 0.02 | 0.426 | 0.319 | 0.328 | 95 |  |  |  |
| Interleukin-8 | prot-a-749 | 0.023 | 0.501 | 0.502 | 0.518 | 95 |  |  |  |
| C-X-C motif chemokine 5 | prot-a-748 | 0.032 | 0.813 | 0.214 | 0.234 | 95 |  |  |  |
| Endothelin-converting enzyme 1 | prot-a-884 | 0.046 | 0.428 | 0.15 | 0.156 | 95 |  |  |  |
| Erythropoietin | prot-a-967 | 0.001 | 0.146 | 0.401 | 0.369 | 95 |  |  |  |
| Ephrin type-B receptor 6 | prot-a-966 | 0.021 | 0.51 | 0.279 | 0.293 | 95 |  |  |  |
| C-X-C motif chemokine ligand 1 | prot-b-16 | 0.031 | 0.056 | 0.969 | 0.928 | 49 |  |  |  |

1. Wang L-J, Li S-C, Li S-W, et al. Gut microbiota and plasma cytokine levels in patients with attention-deficit/hyperactivity disorder. *Translational psychiatry* 2022;12(1):76. doi: 10.1038/s41398-022-01844-x

2. Donfrancesco R, Nativio P, Borrelli E, et al. Serum cytokines in pediatric neuropsychiatric syndromes: focus on Attention Deficit Hyperactivity Disorder. *Minerva Pediatr* 2021;73(5):398-404. doi: 10.23736/S2724-5276.16.04642-9

3. Darwish AH, Elgohary TM, Nosair NA. Serum Interleukin-6 Level in Children With Attention-Deficit Hyperactivity Disorder (ADHD). *J Child Neurol* 2019;34(2):61-67. doi: 10.1177/0883073818809831

4. Zhang J, Luo W, Li Q, et al. Peripheral brain-derived neurotrophic factor in attention-deficit/hyperactivity disorder: A comprehensive systematic review and meta-analysis. *J Affect Disord* 2018;227:298-304. doi: 10.1016/j.jad.2017.11.012 [published Online First: 2017/11/14]

5. Masi A, Quintana DS, Glozier N, et al. Cytokine aberrations in autism spectrum disorder: a systematic review and meta-analysis. *Molecular psychiatry* 2015;20(4):440-6. doi: 10.1038/mp.2014.59 [published Online First: 2014/06/18]

6. Saghazadeh A, Ataeinia B, Keynejad K, et al. A meta-analysis of pro-inflammatory cytokines in autism spectrum disorders: Effects of age, gender, and latitude. *Journal of psychiatric research* 2019;115:90-102. doi: 10.1016/j.jpsychires.2019.05.019 [published Online First: 2019/05/28]

7. Saghazadeh A, Ataeinia B, Keynejad K, et al. Anti-inflammatory cytokines in autism spectrum disorders: A systematic review and meta-analysis. *Cytokine* 2019;123:154740. doi: 10.1016/j.cyto.2019.154740 [published Online First: 2019/06/23]

8. Liu SH, Shi XJ, Fan FC, et al. Peripheral blood neurotrophic factor levels in children with autism spectrum disorder: a meta-analysis. *Scientific reports* 2021;11(1):15. doi: 10.1038/s41598-020-79080-w [published Online First: 2021/01/10]

9. Fraguas D, Díaz-Caneja CM, Rodríguez-Quiroga A, et al. Oxidative Stress and Inflammation in Early Onset First Episode Psychosis: A Systematic Review and Meta-Analysis. *Int J Neuropsychopharmacol* 2017;20(6):435-44. doi: 10.1093/ijnp/pyx015

10. Zhou X, Tian B, Han H-B. Serum interleukin-6 in schizophrenia: A system review and meta-analysis. *Cytokine* 2021;141:155441. doi: 10.1016/j.cyto.2021.155441

11. Upthegrove R, Manzanares-Teson N, Barnes NM. Cytokine function in medication-naive first episode psychosis: a systematic review and meta-analysis. *Schizophr Res* 2014;155(1-3):101-08. doi: 10.1016/j.schres.2014.03.005

12. Gallego JA, Blanco EA, Husain-Krautter S, et al. Cytokines in cerebrospinal fluid of patients with schizophrenia spectrum disorders: New data and an updated meta-analysis. *Schizophr Res* 2018;202:64-71. doi: 10.1016/j.schres.2018.07.019

13. Challa F, Seifu D, Sileshi M, et al. Serum level of high sensitive C-reactive protein and IL - 6 markers in patients with treatment-resistant schizophrenia in Ethiopia: a comparative study. *BMC Psychiatry* 2021;21(1):428. doi: 10.1186/s12888-021-03443-4

14. Al-Asmari AK, Khan MW. Inflammation and schizophrenia: alterations in cytokine levels and perturbation in antioxidative defense systems. *Hum Exp Toxicol* 2014;33(2):115-22. doi: 10.1177/0960327113493305

15. Malmqvist A, Schwieler L, Orhan F, et al. Increased peripheral levels of TARC/CCL17 in first episode psychosis patients. *Schizophr Res* 2019;210:221-27. doi: 10.1016/j.schres.2018.12.033

16. Miller BJ, Herzig K-H, Jokelainen J, et al. Inflammation, hippocampal volume, and cognition in schizophrenia: results from the Northern Finland Birth Cohort 1966. *Eur Arch Psychiatry Clin Neurosci* 2021;271(4):609-22. doi: 10.1007/s00406-020-01134-x

17. El Kissi Y, Samoud S, Mtiraoui A, et al. Increased Interleukin-17 and decreased BAFF serum levels in drug-free acute schizophrenia. *Psychiatry Res* 2015;225(1-2):58-63. doi: 10.1016/j.psychres.2014.10.007

18. Orhan F, Schwieler L, Fatouros-Bergman H, et al. Increased number of monocytes and plasma levels of MCP-1 and YKL-40 in first-episode psychosis. *Acta Psychiatr Scand* 2018;138(5):432-40. doi: 10.1111/acps.12944

19. Wu JQ, Chen DC, Tan YL, et al. Altered interleukin-18 levels are associated with cognitive impairment in chronic schizophrenia. *J Psychiatr Res* 2016;76 doi: 10.1016/j.jpsychires.2016.01.013

20. Li H, Zhang Q, Li N, et al. Plasma levels of Th17-related cytokines and complement C3 correlated with aggressive behavior in patients with schizophrenia. *Psychiatry Res* 2016;246:700-06. doi: 10.1016/j.psychres.2016.10.061

21. Xiu MH, Lin CG, Tian L, et al. Increased IL-3 serum levels in chronic patients with schizophrenia: Associated with psychopathology. *Psychiatry Res* 2015;229(1-2):225-29. doi: 10.1016/j.psychres.2015.07.029

22. Dini AA, Wang P, Ye DQ. Serum Adiponectin Levels in Patients With Systemic Lupus Erythematosus: A Meta-analysis. *Journal of clinical rheumatology : practical reports on rheumatic & musculoskeletal diseases* 2017;23(7):361-67. doi: 10.1097/rhu.0000000000000580 [published Online First: 2017/09/25]

23. Shen HH, Fan Y, Wang YN, et al. Elevated Circulating Interleukin-17 Levels in Patients with Systemic Lupus Erythematosus: A Meta-analysis. *Immunological investigations* 2020;49(6):662-75. doi: 10.1080/08820139.2019.1699107 [published Online First: 2019/12/19]

24. Robak E, Woźniacka A, Sysa-Jedrzejowska A, et al. Serum levels of angiogenic cytokines in systemic lupus erythematosus and their correlation with disease activity. *European cytokine network* 2001;12(3):445-52. [published Online First: 2001/09/22]

25. Mao YM, Zhao CN, Liu LN, et al. Increased circulating interleukin-8 levels in systemic lupus erythematosus patients: a meta-analysis. *Biomarkers in medicine* 2018;12(11):1291-302. doi: 10.2217/bmm-2018-0217 [published Online First: 2018/12/05]

26. Bae SC, Lee YH. Circulating macrophage migration inhibitory factor levels and its polymorphisms in systemic lupus erythematosus: A meta-analysis. *Cellular and molecular biology (Noisy-le-Grand, France)* 2017;63(10):74-79. doi: 10.14715/cmb/2017.63.10.12 [published Online First: 2017/11/04]

27. Resende AL, Elias RM, Wolf M, et al. Serum levels of fibroblast growth factor 23 are elevated in patients with active Lupus nephritis. *Cytokine* 2017;91:124-27. doi: 10.1016/j.cyto.2016.12.022 [published Online First: 2017/01/08]

28. Denny MF, Thacker S, Mehta H, et al. Interferon-alpha promotes abnormal vasculogenesis in lupus: a potential pathway for premature atherosclerosis. *Blood* 2007;110(8):2907-15. doi: 10.1182/blood-2007-05-089086 [published Online First: 2007/07/20]

29. Ding J, Su S, You T, et al. Serum interleukin-6 level is correlated with the disease activity of systemic lupus erythematosus: a meta-analysis. *Clinics (Sao Paulo, Brazil)* 2020;75:e1801. doi: 10.6061/clinics/2020/e1801 [published Online First: 2020/10/22]

30. Llorente L, Richaud-Patin Y, García-Padilla C, et al. Clinical and biologic effects of anti-interleukin-10 monoclonal antibody administration in systemic lupus erythematosus. *Arthritis and rheumatism* 2000;43(8):1790-800. doi: 10.1002/1529-0131(200008)43:8<1790::Aid-anr15>3.0.Co;2-2 [published Online First: 2000/08/16]

31. Tang Y, Xie H, Chen J, et al. Activated NF-κB in bone marrow mesenchymal stem cells from systemic lupus erythematosus patients inhibits osteogenic differentiation through downregulating Smad signaling. *Stem cells and development* 2013;22(4):668-78. doi: 10.1089/scd.2012.0226 [published Online First: 2012/08/18]

32. Dellalibera-Joviliano R, Reis ML, Donadi EA. Kinin system in lupus nephritis. *International immunopharmacology* 2001;1(9-10):1889-96. doi: 10.1016/s1567-5769(01)00109-6 [published Online First: 2001/09/20]

33. Li HM, Zhang TP, Leng RX, et al. Plasma/Serum Leptin Levels in Patients with Systemic Lupus Erythematosus: A Meta-analysis. *Archives of medical research* 2015;46(7):551-6. doi: 10.1016/j.arcmed.2015.09.005 [published Online First: 2015/10/11]

34. Xiang M, Feng Y, Wang Y, et al. Correlation between circulating interleukin-18 level and systemic lupus erythematosus: a meta-analysis. *Scientific reports* 2021;11(1):4707. doi: 10.1038/s41598-021-84170-4 [published Online First: 2021/02/27]

35. González-Serna D, Ortiz-Fernández L, Vargas S, et al. Association of a rare variant of the TNFSF13B gene with susceptibility to Rheumatoid Arthritis and Systemic Lupus Erythematosus. *Scientific reports* 2018;8(1):8195. doi: 10.1038/s41598-018-26573-4 [published Online First: 2018/05/31]

36. Lee JM, Kronbichler A, Park SJ, et al. Association between Serum Matrix Metalloproteinase- (MMP-) 3 Levels and Systemic Lupus Erythematosus: A Meta-analysis. *Disease markers* 2019;2019:9796735. doi: 10.1155/2019/9796735 [published Online First: 2019/08/10]

37. Lee YH, Bae SC. Circulating leptin level in rheumatoid arthritis and its correlation with disease activity: a meta-analysis. *Z Rheumatol* 2016;75(10):1021-27. doi: 10.1007/s00393-016-0050-1 [published Online First: 2016/01/29]

38. Zhao YL, Wu J, Zhang TP, et al. Circulating Insulin-like Growth Factor-1 Levels in Patients with Rheumatoid Arthritis: A Meta-analysis. *Curr Pharm Des* 2019;25(10):1091-98. doi: 10.2174/1381612825666190319124009 [published Online First: 2019/03/21]

39. Buondonno I, Rovera G, Sassi F, et al. Vitamin D and immunomodulation in early rheumatoid arthritis: A randomized double-blind placebo-controlled study. *PLoS One* 2017;12(6):e0178463. doi: 10.1371/journal.pone.0178463 [published Online First: 2017/06/06]

40. Koch AE, Kunkel SL, Harlow LA, et al. Epithelial neutrophil activating peptide-78: a novel chemotactic cytokine for neutrophils in arthritis. *J Clin Invest* 1994;94(3):1012-8. doi: 10.1172/jci117414 [published Online First: 1994/09/01]

41. Kragstrup TW, Greisen SR, Nielsen MA, et al. The interleukin-20 receptor axis in early rheumatoid arthritis: novel links between disease-associated autoantibodies and radiographic progression. *Arthritis Res Ther* 2016;18:61. doi: 10.1186/s13075-016-0964-7 [published Online First: 2016/03/13]

42. Kim W, Min S, Cho M, et al. The role of IL-12 in inflammatory activity of patients with rheumatoid arthritis (RA). *Clin Exp Immunol* 2000;119(1):175-81. doi: 10.1046/j.1365-2249.2000.01095.x [published Online First: 1999/12/22]

43. Qiao YC, Chen YL, Pan YH, et al. The change of serum tumor necrosis factor alpha in patients with type 1 diabetes mellitus: A systematic review and meta-analysis. *PloS one* 2017;12(4):e0176157. doi: 10.1371/journal.pone.0176157 [published Online First: 2017/04/21]

44. Qiao YC, Shen J, Hong XZ, et al. Changes of regulatory T cells, transforming growth factor-beta and interleukin-10 in patients with type 1 diabetes mellitus: A systematic review and meta-analysis. *Clinical immunology (Orlando, Fla)* 2016;170:61-9. doi: 10.1016/j.clim.2016.08.004 [published Online First: 2016/08/09]

45. Vaseghi H, Sanati MH, Jadali Z. T-helper Cell Type-1 Transcription Factor T-Bet Is Down-regulated in Type 1 Diabetes. *Iranian journal of allergy, asthma, and immunology* 2016;15(5):386-93. [published Online First: 2016/12/06]
